# GLADX: An Automated Approach to Analyze the Lineage-Specific Loss and Pseudogenization of Genes

**DOI:** 10.1371/journal.pone.0038792

**Published:** 2012-06-18

**Authors:** Jacques Dainat, Julien Paganini, Pierre Pontarotti, Philippe Gouret

**Affiliations:** Aix-Marseille Université Laboratoire d’Analyse, Topologogie, Probabilités (LATP) UMR–CNRS 7353 équipe Evolution Biologique & Modélisation, Marseille, France; Barnard College, Columbia University, United States of America

## Abstract

A well-established ancestral gene can usually be found, in one or multiple copies, in different descendant species. Sometimes during the course of evolution, all the representatives of a well-established ancestral gene disappear in specific lineages; such gene losses may occur in the genome by deletion of a DNA fragment or by pseudogenization. The loss of an entire gene family in a given lineage may reflect an important phenomenon, and could be due either to adaptation, or to a relaxation of selection that leads to neutral evolution. Therefore, the lineage-specific gene loss analyses are important to improve the understanding of the evolutionary history of genes and genomes. In order to perform this kind of study from the increasing number of complete genome sequences available, we developed a unique new software module called GLADX in the DAGOBAH framework, based on a comparative genomic approach. The software is able to automatically detect, for all the species of a phylum, the presence/absence of a representative of a well-established ancestral gene, and by systematic steps of re-annotation, confirm losses, detect and analyze pseudogenes and find novel genes. The approach is based on the use of highly reliable gene phylogenies, of protein predictions and on the analysis of genomic mutations. All the evidence associated to evolutionary approach provides accurate information for building an overall view of the evolution of a given gene in a selected phylum. The reliability of GLADX has been successfully tested on a benchmark analysis of 14 reported cases. It is the first tool that is able to fully automatically study the lineage-specific losses and pseudogenizations. GLADX is available at http://ioda.univ-provence.fr/IodaSite/gladx/.

## Introduction

Essential genes, such as housekeeping genes or genes involved in interaction networks, remain stable during evolution due to their central biological role and tend to evolve under purifying selection [1]–[3]. The more the gene is important, the more it tends to be universally conserved. Unlike the gene losses due to functional redundancy after gene duplication [4], the lineage-specific losses of well-established genes may reflect significant changes [5]–[7]. Two mechanisms might describe the losses of well-established genes: i) losses that are not linked to environmental shifts, but to the presence of other genes in the genome that can fulfill the original functions, and ii) losses linked to environmental shifts, and that can be either produced by genetic drift with no selection (i.e. they encode functions that are no longer useful), or by adaptive negative selection (i.e. the maintenance of functions that generate handicaps). The counterintuitive concept that gene losses may be an important driver of evolutionary change via adaptive changes was named the “less is more” hypothesis [8]. The lineage-specific gene losses of well-established genes can be due to deletion events or to pseudogenizations. This kind of pseudogenes is called unitary pseudogenes [9]. After a certain time it is not possible to differentiate between the two cases. Indeed, once that a gene was deactivated by a deleterious mutation it becomes a pseudogene which evolves free from selective constraints and undergoes a progressive erosion of the signal by accumulation of numerous further mutations until the footprint of the original sequence becomes unrecognizable in the genome among the non-coding signal. When pseudogenization is not too old, mutations of this kind are still observable.

An extensive orthology/paralogy assessment is necessary to identify gene losses between different species. Recent analyses show that phylogeny-based methods are generally more reliable than similarity-based approaches. Phylogeny-based methods to detect relationships between sequences use reconciliation of species and gene trees to infer speciations and duplications; and to visualize the loss events. These methods have been studied since the 1970s [10]–[14]. Gene disappearances leading to extinction of functions have been identified in specific gene families and allowed the discovery of unitary pseudogenes [5]–[7], [15]. Whole genomes sequencing has been made technically possible to study by comparative analyses of lineage-specific losses, bringing to light this major evolutionary process [16]–[18]. The increasing availability of complete genome sequences makes possible the investigation of these losses at a large scale, find co-elimination of functionally-connected groups of genes [17], and thus consider co-losses in different lineages. Many comparative analysis methods were developed to study the lineage-specific losses. The most commonly used method is the creation of orthologous groups by reciprocal blasts and inference of presence and absence of orthologs on a phylogenetic tree [19]–[23]. Detection of these losses can also be performed by reading phylogenies [24]. In addition, other methods are specific to analyze the unitary pseudogenes [15], [25]–[27] using the conserved synteny of neighboring genes. Totally automated methods to analyze lineage-specific losses and pseudogenizations are still lacking yet.

The aim of this study being the automation of the lineage-specific losses analyses, we developed a dedicated module that is part of the multi-agent system DAGOBAH [28] that we named **G**ene **L**oss **A**nalyzer **D**AGOBAH e**X**tension (GLADX). Each GLADX step was inspired by human expertise and engineered to closely mimic its characteristics. From a given sequence as input GLADX performs a gene phylogeny based on protein alignment of selected species-set, and by a tree reading method, detects the putative lineage-specific losses of the gene family. For all the candidate species to a lineage-specific loss, the module performs a comprehensive study to confirm losses and search pseudogenes. By a re-annotation systematic method of orthologous sequences recovered in genomes, GLADX is able to find and differentiate pseudogenes and intact but un-annotated genes present in databases and that would have been missed during previous rounds of genome annotation. The distinction between novel genes and pseudogenes is first performed at protein level by comparing the protein predictions, and complemented at nucleotide level when quality of sequences allow it. GLADX offers deeper insights on the pseudogenization, thanks to step of ancestral sequences reconstruction and to the analysis of mutations that occurred during evolution. GLADX offers the possibility to launch simultaneously several studies. For each sequence given as input it automatically resolves in the selected species-set all the events that occurred during the course of evolution of gene family. The evolutionary aspect is given based on gene phylogenies, ancestral sequence reconstruction, and a parsimony algorithm to locate the detected events. All events and traits found by GLADX are summarized and pinpointed on a user friendly species tree. The used innovative approach combines the quality resolution of phylogeny-based homology relations, a search at protein and nucleotide level, an evolutionary view of events, and total automation thereby substantially improving the set of tools which were available yet.

## Methods

The DAGOBAH framework [28] in which GLADX is implemented uses the Prolog and Java languages. DAGOBAH framework is a set of agents running in parallel, sharing persistent results and that can communicate between each other and with external software platforms such as Ensembl, NCBI and FIGENIX [29]–[32]. An agent is like standalone software but belongs to an applicative context. DAGOBAH is designed to automatically predict and localize phylogenetically all the genetic events that occurred during the evolutionary history of genes. Within DAGOBAH, the GLADX module features 13 agents, some of which are not specific to GLADX and can be re-used in other contexts (i.e. gene phylogeny-building, ancestral sequence reconstruction, genes prediction). GLADX is not a standalone tool and depends to the established DAGOBAH framework.

The main purpose of GLADX is to automatically detect the lineage-specific loss, pseudogenization or presence of orthologous genes from a protein sequence in FASTA format given as input. In order to perform a reliable study, GLADX needs to use a database containing the complete proteomes and/or genomes of the desired set of species. The choice of species used by GLADX during studies needs to be specified (Text S1, A). A binary species tree containing these species needs also to be defined in GLADX. This binary species tree and the branch lengths can be easily changed by users. It is used at tree reconciliation step in gene phylogeny pipelines to deduce duplication events, and at different GLADX steps to define the relatedness of species. It is also used at the end of studies to perform an annotated species tree on which are summarized all the found events. It is expected that a change of this species tree can modify the detected losses and the placement of the reconstructed evolutionary events. (A view of the species tree implemented in the downloadable GLADX version is available, Text S3). As studies are performed, new data obtained after each important step is saved in a Report file and in an ontological database (Figure S1). The ontological records make it possible to restart at the last step performed so as to continue a study after an accidental stop. They allow also storing important data that may subsequently prove useful to a biologist or computational biologist.

Currently the GLADX version available for download is configured to study 22 *Chordates* species from Ensembl V57 and allows studying the pseudogenization (correspond to analyses in complete mode). The pseudogenization analyses require the genomes and proteomes sequences of studied species in GLADX. In this mode, a maximum of 51 *Chordates* from Ensembl version 48 to 58 can be analyzed. When studies are launched without research of pseudogenes (corresponds to simple mode), only proteomes are necessary. In this case the proteomes sequences can come from any database, and the number of species used is not limited. The addition of species requires completing the species tree used by GLADX.

**Figure 1:**
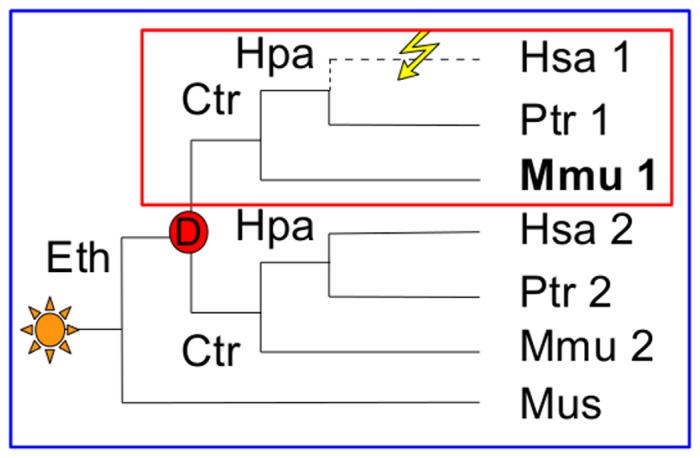
Approaches for detecting orthology and loss events: example of gene trees. Gene appearance detected by the phylogeny is depicted by the yellow star and the red circle represents gene duplication event. In bold is the gene used as input to build the tree. Based on the orthologous group from the Eth ancestor (blue frame) no lineage-specific loss is evidenced because each species has an ortholog to Mus. Based on the Ctr ancestor orthologous group, there are two orthologous groups, but only one group has an lineage-specific loss (red frame). Note that all abbreviations concerning species name and their ancestors are provided in Figure 6.

To study lineage-specific losses, we developed an approach that detects an orthologs group stemming from a selected common ancestral species. It makes possible to determine the ancestor from which a gene is established and to find among species stemming from this ancestor, those having no representative of the ancestral gene. GLADX considers the orthologous group defined by the sub-tree which has a speciation node on the selected ancestor and containing the input reference gene. By default, if a speciation node in the defined ancestor does not exist, the next speciation node in the leaves direction is used. In a first example (Figure 1, the blue frame), the ancestor considered is the LCA of *Eutheria*. All genes present in the tree form an *Eutheria* orthologous group, because they are co-orthologs to the *Mus* gene. Despite possible loss of genes (here a human gene), no lineage-specific loss of the *Eutheria* ancestral gene will be detected because one representative is present as counterpart in each species of the set. In the second case, the LCA of *Catarrhini* was selected as ancestor. The gene should be present in *Homo*, *Pan* and *Macaca*. There are two sub-trees, but only one containing the reference gene used as input to build the tree will be analyzed. Here, this reference sequence is *Mmu1*. In the found orthologous group, the human gene is absent from the gene phylogeny. This is a *Homo* lineage-specific gene loss of a gene established since the LCA of *Catarrhini* (Figure 1, the red frame). A GLADX agent can be activated to systematically scan all the nodes along the lineage starting from the selected ancestor and leading to the used reference. Each node corresponding to the establishment of a new sub-family (a speciation node after a duplication event) is studied to find the lineage-specific losses of selected species (Text S1, G). This agent is available for all studies performed by GLADX, whatever the number of species used. By default, this option is not activated to allow choosing the kind of lineage specific losses searched. As example, to find *Vertebrate* specific losses (gene that was present in the *Vertebrates* ancestor, and subsequently lost in species of the phylum), the ancestor determined by the user should be those of *Vertebrates*. Several parameters can change how GLADX behaves and can modulate its execution (Text S1). By default lineage-specific losses of genes established since the LCA of *Eutelostomi* are analyzed (Text S1, B). The method implemented in GLADX is developed below in further detail through the different main steps.

**Figure 2:**
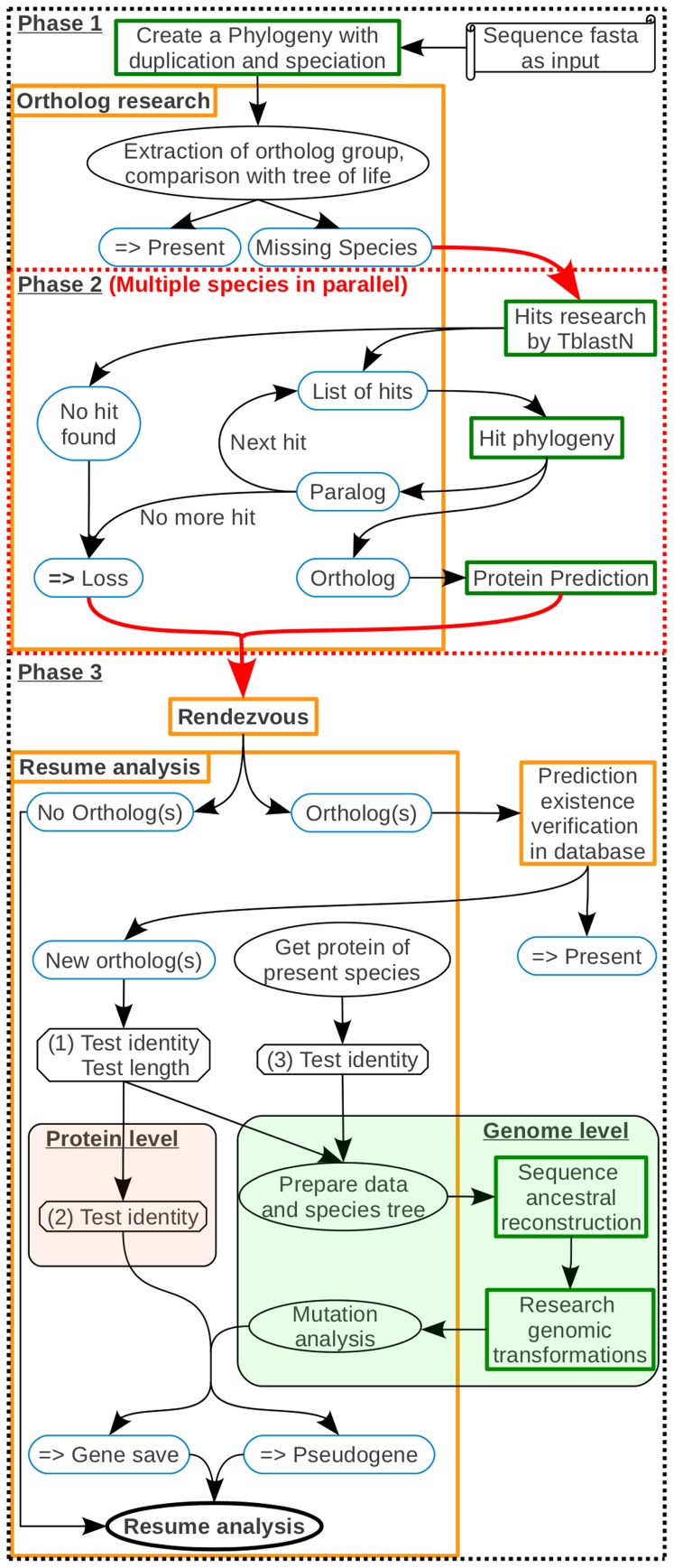
Method for identifying lineage-specific gene losses and pseudogenes. Parchment illustrates the necessary data for starting a study. Rectangles correspond to agents with their descriptions. The agents launching pipelines are in the green frame. Blue round-edge rectangles highlight essential results. Those with horizontal double arrows are final conclusions. Ellipses are a state or an action. Octagons consist in tests or analyses necessary to define the follow-up on the study. Arrows show the study pathway. Phase 1: Detection of species in which orthologs are missing. Phase 2: Parallel studies screening each orthologous sequence for each missing species. Phase 3: The red arrow is a factorization of the different species studied. The rendezvous agent aims at waiting that all species targeted in the study have been done, before continuing. During this phase, GLADX tries to find the reasons explaining why the orthologs were missing. Depending on the sequences conservation, saved orthologs are analyzed either at protein level, or at nucleotide level, or the both.

### The Gene Phylogeny as Starting Point

A FASTA format protein sequence is given as input. The first step builds a gene phylogeny based on protein alignment of species list. The gene phylogeny is built using an automated gene phylogeny pipeline available on the FIGENIX platform [32] connected to DAGOBAH. FIGENIX gives gene phylogenies with the speciation and duplication events annotated using the Forester detection algorithm [14] that compares a consensus tree obtained with the species tree defined in GLADX.

### Detection of Species that have no Orthologs

From the gene phylogenetic tree, the PhyloPattern API [33] integrated to DAGOBAH, is used by GLADX to search patterns and so to automatically detect the largest orthologs group containing the initial query sequence, according to the user’s choice of phylum to be studied. Cross-comparing the species-set present in the group of orthologous sequences detected against the list of the origin studied species-set, makes it possible to identify species which possess no orthologous gene. These species are candidates for lineage-specific gene loss.

A **simple** mode exists for GLADX, and avoids the verification of putative losses detected at this step. It jumps directly at final steps to display the presence and absence of an orthologous gene on leaves of the final phylogenetic view. Via a Dollo-like parsimony method, GLADX infers the loss events on the lineages in which they occurred. This mode is better suited to study old losses. Indeed, even if the loss comes from a pseudogenization event, after a long evolutionary time it is not necessary to search an ancient pseudogenized sequence whose traces should have erased under neutral evolution [34]. This mode optimizes the computation time and may be used with any protein sequence database.

With **complete** mode (by default), each putative lineage-specific loss is confirmed by a deeper analysis that is developed subsequently. This deeper analysis allows to find pseudogenes and novel genes that will be analyzed both at the protein and nucleotide level. Currently, GLADX was implemented to use the proteomics and genomics databases from Ensembl for studies carried out on this mode, but other databases can be integrated by short developments.

### Screening for Orthologous Sequences

To check that a gene is in fact completely absent from the genome sequence of a candidate species, GLADX scans the gene phylogeny and takes an orthologous protein of the closest species. GLADX uses this orthologous protein as a reference to find putative homologous sequences within the genome of the candidate species, using the TBLASTN algorithm [35]. If no hit is detected, a gene loss is inferred; but if putative homologous sequences are found, GLADX checks among them for orthologs. To check orthology of the putative homologous sequences, a phylogenetic approach is used again. At this step, to get the best sequence signal as possible to produce reliable gene phylogenies, an implemented method screens the TBLASTN hits, searching those that are tidy, carried by the same chromosome, sharing an identical direction, and that are close to each other, to concatenate them together. Afterwards, the created sequences are sorted by blast score decreasing order in a list (Text S1, C). Gene phylogenies from hits are built one after another, until one hit is found orthologous to at least one protein of the orthologous group defined at the first step. If no orthologous sequence stands out from candidate sequences, it then infers a gene loss. But if an ortholog is retrieved, the software still have to confirm whether this orthologous sequence corresponds to an already annotated gene eliminated by the gene phylogeny [31], an un- or mis-annotated gene, or to a pseudogene.

### Analysis of Saved Orthologs

Coding sequences are better conserved at protein level than at nucleotide level. To avoid running an analysis at nucleotide level when observations are made impossible by high involved divergence, the analysis of each recovered orthologous sequence is first done at protein level. For this purpose, a protein prediction is built from a large piece of DNA containing the orthologous signal found by TBLASTN. Predictions are built using a pipeline embedded to the GenePredix pipeline [31], modified to take a reference protein sequence and a DNA sequence as inputs. Its aim is to predict the most similar protein to the reference protein from the DNA sequence. Among the orthologous sequences group defined at the first step, GLADX chooses as reference a protein of a species that is phylogenetically closer to the species from which the DNA sequence studied comes from. Once the prediction is done, GLADX systematically screens the database used to test whether a similar gene in the genome has already been described at the same location (Text S1, F). It allows verifying that the found gene does not correspond to a gene which is missing in the gene phylogeny of the study starting point. When a similar gene is already present, GLADX concludes that no lineage-specific loss occurred in the studied species. At this point, GLADX tests the orthology annotation of the regained gene, and saves the knowledge of this new orthology if the information was missing in the used database. Whereas no similar gene was described at the location, GLADX concludes that the predicted protein was never described. Then, an analysis will be performed to discern if the predicted protein comes from a putative gene, unless the orthologous nucleotide sequence found is a pseudogene and the predicted protein should not exist. To choose the depth of the next analysis (protein or nucleotide level), a test of length and similarity of the orthologous protein sequence found by TBLASTN and of the protein prediction that followed from it, is performed, using the known orthologous proteins as reference (Figure 2, test 1; Text S1, D). The similarity test is performed using the Needleman-Wunsch algorithm [36]. The length ratio, expressed in percentages, is calculated using the length of the reference protein. When the length and the similarity percentage of both tested orthologous protein sequences are under the user-defined thresholds, the study remains at protein level; otherwise, when the features of one of analyzed orthologous protein sequences exceeds the user-defined thresholds, a study is performed at nucleotide level. The reason both protein sequences are tested is that the prediction may not be sufficient and worse than the “hit” sequence when the DNA sequence has nonsense mutations. Indeed, if nonsense codons are present in the nucleotide sequence, the prediction must avoid them correctly, by moving the prediction start or end, splicing them into introns, or changing the reading frame. Therefore the predicted protein will be shortened or no protein will be predicted. Alternatively, blast hits are unhampered by nonsense codons. There they have a low impact on sequence recovered.

**Figure 3:**
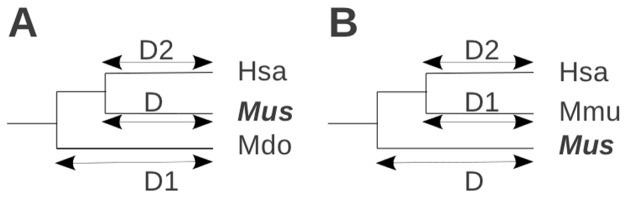
Test used at protein level to conclude on putative gene or putative pseudogene. The species where the protein is tested is highlighted in bold type. Other species have known proteins. *D1* and *D2* are age of divergence, in millions of years. Note that all abbreviations concerning species name and their ancestors are provided in Figure 6 (A) The species in which the protein is analyzed is surrounded by species having different LCAs. If the sequence identity is higher between *Mus* and *Hsa* as compared to *Mus* and *Mdo* thus, we can conclude on a putative gene; otherwise on a putative pseudogene. (B) If all species with known protein have the same LCA as the species under investigation, a calculation step is necessary. *Value1* is the percent identity between *Mus* and *Hsa*. *Value2* is the percent identity between *Mmu* and *Hsa*. A minimum relative threshold is calculated by multiplying *D2* distance by the similarity percentage *Value2* and by dividing the total by the distance *D*. If the similarity percentage *Value1* is superior to the minimum relative threshold calculated, we conclude on a putative gene; otherwise, we conclude on a putative pseudogene.

### Analysis at Protein Level

In cases involving a protein-level analysis, GLADX uses the best protein predicted from the DNA of the orthologous TBLASTN hits found. To check whether or not each retrieved orthologous sequence is a putative pseudogene, it is necessary to check whether the conservation of the predicted protein is consistent with the divergence time observed among its orthologous sequences (Figure 2, test 2). We assume that in an orthologous group the protein sequence conservation should remain proportional to the divergence time between species that carry them. This consistency can be tested in two ways depending on species that possess known orthologous proteins. In the first case, it exists two species which do not share the same LCA with the species in which the protein sequence is being investigated (Figure 3, A). The test will be positive when the similarity percentage between the recovered orthologous protein sequence and the less-diverging protein is higher than the similarity percentage with the protein from the more divergent species. In the second case, all available orthologous proteins of the study come from species that shares the same LCA with the studied species (Figure 3, B). The divergence times between the protein under investigation and the known proteins are identical. In this case, an average of decreasing of similarity percentage per million years according to the divergence observed between the known proteins is calculated. The test will be positive when the similarity percentage of the tested protein with a known protein is higher than the minimum similarity percentage expected according to their divergence time. When the conservation of the predicted protein gives a consistent result, we conclude on the putative existence of that orthologous gene in the candidate genome under study; otherwise, we conclude that the orthologous sequence undergoes a pseudogenization (Figure 2, test 2).

**Figure 4:**
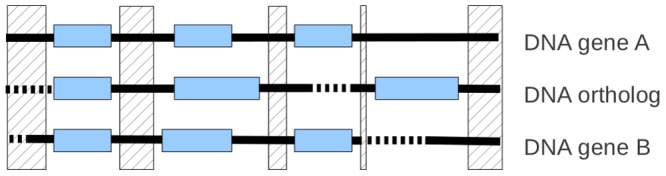
Processing of an alignment and conservation of informative signal of sequences. The hatched area is deleted area, blue boxes are exons (exons of retrieved orthologs come from prediction), bold black lines are DNA, and bold dotted lines are gaps.

### Analysis at Nucleotide Level

**Figure 5:**
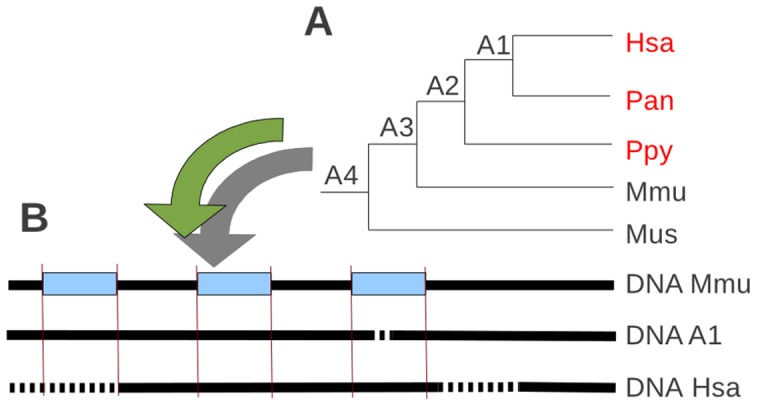
Example of processing sequences from a multiple sequences alignment and their ancestral sequences reconstructed. (A) Tree of species used for the reconstruction step. Species in red have sequence orthologs retrieved by GLADX. *A1*, *A2*, etc. correspond to ancestors reconstructed by Ortheus from orthologous sequences. Once this ancestral reconstruction is finished, the scan step is launched. (B) Sequences considered for an analysis at nucleotide level. Five scans will be carried out (*Hsa* vs *A1*, *Pan* vs *A1*, *Ppy* vs *A2*, *A1* vs *A2*, and *A2* vs *A3*). Only *Hsa* against *A1* is described here. The exon projection is necessary to each scan. Bold black lines are DNA, bold dotted lines are gaps, and red lines are projections from reference exons. Note that all abbreviations concerning species name and their ancestors are provided in Figure 6.

When the orthologous TBLASTN hit or the orthologous protein prediction successfully passes the first test (Figure 2, test 1), an analysis at the nucleotide level is performed in order to decipher and unravel the genetic events that affected the sequence during evolution. All recovered orthologous sequences that must be analyzed at nucleotide level, are tested together in one step. Together, the DNA sequence of all the recovered orthologous sequences, and those from known orthologous genes that are found not too divergent (Figure 2, test 3, Text S1, D), are sent to an ancestral sequence reconstruction-dedicated pipeline. No more as one sequence by species is used. This pipeline breaks down into two steps. Step one uses “LaganM” [37], a multiple aligner based on the CHAOS local alignment tool that combines speed and high accuracy for large sequences. It aligns the orthologous DNA sequences and compacts the coding sequences areas (Figure 4). The compaction process consists in only keeping the coding region signal in order to cut computation time and improve the quality of the subsequent processing work. Step two uses “Ortheus” to perform ancestral sequence reconstruction from the sequence alignment [38]. It builds a phylogenetic tree, and using efficient stochastic graph-based dynamic programming methods, it builds a multiple-sequence ancestor alignment, which contains explicit ancestor sequences for every node of the phylogeny. Identifying the ancestral sequence is highly valuable for revealing the step-by-step series of genetic events occurring during the gene evolution in a lineage. However, unlike routine alignments where indels are not interpreted, this multiple-sequence ancestor alignment is able to differentiate insertions from deletions. It is important to consider that to take in account the phenomenon of allele sorting [39] and to follow the real gene history, “Ortheus” is configured to reconstruct the ancestral sequences following its proper phylogeny, estimated by neighbor joining method with HKY model. Despite one sequence by species is used, the outcome may be a tree that is different to the species tree (a parameter can be added to force reconstruction based on a selected phylogeny). Working from this alignment each sequence, requiring an analysis, and its closest ancestral sequence built are sent to an agent dedicated to reveal the genetic mutations that appear between the ancestral sequence and the sequence analyzed (ancestral or contemporary). As we do not know the exons on the sequence retrieved and also in the reconstructed ancestor, the agent uses the exon positions inferred from a known gene and present in the alignment (Figure 5). Sequence comparison then makes it possible to test: i) the presence or absence of the start and/or final nonsense codons; ii) the occurrence of nonsense, insertion and/or deletion mutations in the open reading frame; iii) the loss and the modification of splice sites; and iv) the loss of exons. Thus, if the analysis does not return any degenerate mutations, we conclude on the putative existence of this orthologous gene in the genome of the candidate species; otherwise, it concludes on a pseudogenization.

**Figure 6:**
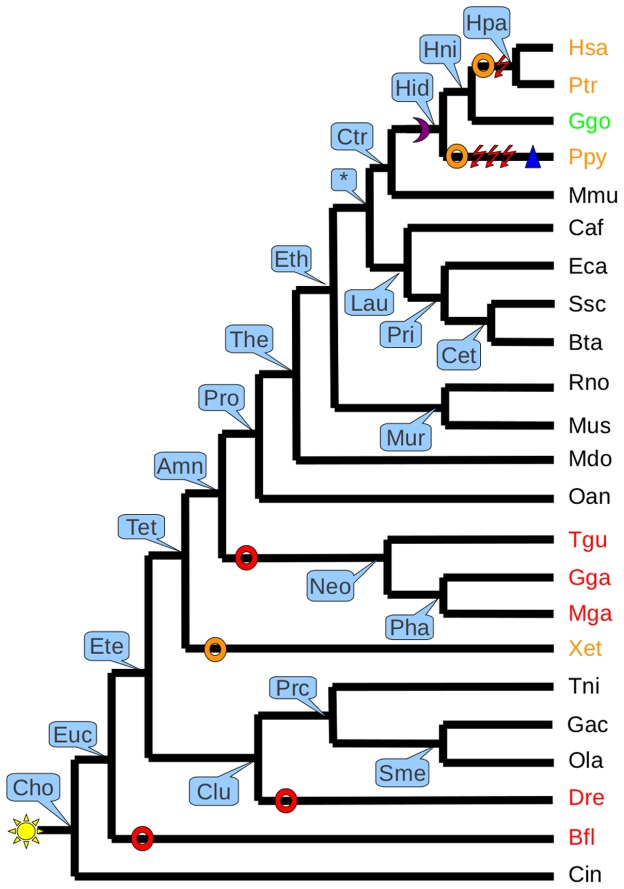
Summary of events occurred during evolution of *Acyl3* in 23 species. Each ancestor is indicated in blue frame by their abbreviation. All events are pinpointed on the specific branches. The asterisk indicates ancestor subject of controversy. Red rings show loss events; Orange rings show pseudogenization events; Magenta moon is a splice site mutation; Red flashes represent nonsense codon appearance; Blue triangle is insertion. The yellow star is the point of gene appearance in the phylogenetic tree. The species in red are species that have lost the gene. The species in orange have an orthologous sequence considered as pseudogene, and species in green is species where the recovered gene is considered as potentially intact. The complete name of species and ancestors are described in the following abbreviation paragraph. Name abbreviation of species: Bfl: Branchiostoma floridae; Bta: Bos taurus; Caf: Canis familiaris; Cin: Ciona intestinalis; Dre: Danio rerio; Eca: Equus caballus; Gac: Gasterosteus aculeatus; Gga: Gallus Gallus; Ggo: Gorilla gorilla; Hsa: Homo sapien; Mdo: Monodelphis domestica; Mga: Meleagris gallopavo; Mmu: Macaca mulatta; Mus: Mus musculus; Oan: Ornithorhynchus anatinus; Ola: Oryzias latipes; Ptr: Pan troglodytes; Ppy: Pongo pygmaeus Abelii; Rno: Rattus norvegicus; Ssc: Sus scrofa; Tgu: Taeniopygia guttata; Tni: Tetraodon nigroviridis; Xet: Xenopus tropicalis; Name abbreviation of ancestors: Amn: Amniota; Cet: Cetartiodactyla; Cho: Chordata; Clu: Clupeocephala; Ctr: Catarrhini; Ete: Euteleostomi; Eth: Eutheria; Euc: Euchordata; Hid: Hominidae; Hni: Homininae; Hpa: Homo/Pan ancestor; Lau: Laurasiatheria; Mur: Murinae; Neo: Neognathae; Pha: Phasianidae; Pri: Perissodactyla; Prc: Percomorpha; Pro: Prototheria; Sme: Smegmamorpha; Tet: Tetrapoda; The: Theria;

### Synthesis of Results

The obtained results are summarized on the species tree used in GLADX on which all information is highlighted. On one hand, for each species, the state of presence of one representative of the gene-of-interest family is indicated on leaves of the tree by the character Present, Saved, Pseudogene or Lost. These characters may give insight about the current state of the function associated to the family of the gene-of-interest in the species studied. Sankoff parsimony [40], [41] is used to highlight the ancestral and derived traits, making it possible to highlight the evolutionary aspect by defining the event occurrence dates of lineage-specific pseudogenization and loss of gene. Moreover, it allows calculating the ancestor from which the studied gene family seems to be born. On the other hand, the mutations found at nucleotide level are displayed directly on the phylogenetic tree. These genetic mutations are directly observable in an evolutionary dimension, as they show branch-by-branch the mutations found at nucleotide level, which occurred since the last ancestor having the gene intact, until the contemporary sequences that were investigated (Figure 6).

## Results

To demonstrate the efficiency of GLADX, we used it in complete mode on 23 Chordate species (see Figure 6). It was first benchmarked on 14 cases of unitary pseudogenes or gene losses in the human lineage described in the literature. This benchmark is a positive control demonstrating the tool’s capacity to detect and correctly analyze the lineage-specific events occurring during the evolution of orthologous genes. We then performed a negative control to verify that GLADX does not overpredict gene loss or pseudogenization events. We obtained convincing results, as described below.

The 14 results obtained during the positive control test-runs are described in *Table S1* and summarized in *Table 1*. They are also available on the IODA (Interface for Ontological Data Analysis) user-friendly website (http://ioda.univ-provence.fr/). From 14 literature cases, our results are in line with results previously published in 13 cases. The events analyzed occurred at different times in the human lineage. Some unitary pseudogenes are human-specific, while others have undergone pseudogenizations that have begun in an ancestor. Our analysis is in fact more complete, as GLADX makes it possible to show what happened in all lineages and species of the dataset.

**Table 1:** Benchmark results of 14 pseudogenes cases described in literature.

Gene symbol	Publications	Agree	Precision	Artifacts highlighted	Comments
2310042E22Rik	[26], [27]	no	/	/	The gene was saved and detected as intact.
Gulo	[5], [26], [27]	yes	–	**	Problem of sensibility, tracks of pseudogenes are not detected
Acyl3	[26], [27]	yes	+	/	/
Uox	[6], [26], [27], [45]	yes	=	/	/
Ctf2	[26], [27], [46]	yes	+	/	/
Nradd	[26], [27], [47]	yes	+	*	/
Nepn	[26], [27], [47]	yes	+	*	/
Mup4	[27], [48]	yes	+	*	/
T2r2	[49]	yes	+	/	Pseudogene was described as polymorphic
Tas2R134	[27], [49]	yes	=	/	Tas2R134 and Tas2R143 *Mus* genes are co-orthologs to human pseudogene
1110012D08Rik	[27]	yes	+	*	/
Gpr33	[26], [27], [47]	yes	+	/	/
Slc7a15	[26], [27], [47]	yes	+	***	Some mutations are not seen due to artifacts
Sult3a1	[42]	yes	–	**	Artifacts leads to a scan in false frame, but the pseudogenization was confirmed manually

The ***Publications*** column indicates references of studies of the literature used as reference for comparison with GLADX results.

The ***Agree*** column contains yes if the case is consistent with the literature results and no when the result is in contradiction.

The ***Precision*** column indicates the quality of results obtained: “-” means low precision, “+” means a better precision, “ = ” means we found exactly the same results, and “/” means it can't be interpreted.

The ***Artifacts*** ** ***highlighted*** column indicates cases where artifacts are present: “*” are cases where GLADX found artifacts in databases (Text S2); “**” means artifacts caused by tools implemented in GLADX, and “/” means no artifact was observed.

The ***Comments*** column indicates some particularities, “/” means no particular comment.

In 2 cases, GLADX found exactly the same results on pseudogenization in the human lineage, and in 9 cases, GLADX identified interesting new information and sometimes further details allowing us to refine the previous descriptions. Furthermore, it identified and described more genetic mutation events, and in 4 cases it was able to date the beginning of pseudogenizations, more ancestrally than those previously described. These refined results were not surprising given that we often used more species in our analysis. It is due also to the fact that the ancestral sequence reconstruction step enables a sharp detail of genetic events that occurred during the evolutionary course, which is a feature that guarantees increased usage of this method in the future. To illustrate this precision gain, take the example of the gene coding for acyltransferase 3 (*Acyl3)* protein [26], which the authors described as an unitary pseudogene in *Homo* and *Pan* due to a common nonsense mutation that appeared in exon seven after *Gorilla* diverged from the human lineage and before the Homo-Pan split. With GLADX, we not only found the mutations already described but also many other hitherto not described mutations (Figure 6). We discovered that a splice mutation appeared before the LCA of *Hominidae* and after *Macaca* diverged from the *Hominidae* lineage that seems to be the first event leading to the pseudogenization. Independently, four nonsense mutations and an insertion of four bases occurred in *Pan* after the Homo-Pan split and one nonsense mutation (previously described) occurred before the LCA of *Homo* and *Pan* and after *Gorilla* diverged from the human lineage. In addition, the analysis also revealed a loss of the *Acyl3* gene in the lineage leading to *Neognathae* from the LCA of *Amniota*. The fact that three species have lost the gene in *Neognathae* reinforces the idea that it is not a sequencing artifact. Loss of this gene also occurred in *Branchiostoma* lineage after the split with the LCA of *Chordata*, and in the *Danio* lineage after the split with the LCA of *Clupeocephala*. We also found a pseudogenization in the *Xenopus* lineage occurring after the split with the LCA of *Tetrapoda*. Indeed, we found an orthologous sequence of the *Mus* gene that was still present in the Xenopus genome, but the signal was too low and it was under the threshold configured to make it analyzable at the nucleotide level. Three possibilities could explain this fact: the first would be a pseudogenization that has begun in the not too distant past, with the result that the signal has not yet been totally erased; the second possibility is that the gene has evolved more rapidly than in other species, making its similarity percentage lower than the average of most other species; and the third explanation may be that another type of event occurred during this gene’s evolution, such as a shuffling, partial gene loss, etc. Neither the losses nor the pseudogenization in *Pongo* and *Xenopus* were described before.

**Figure 7:**
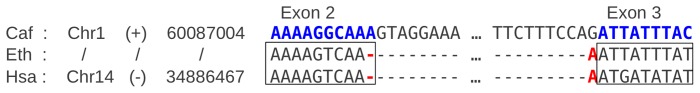
Inherent error generated during alignment processing of *Sult3a1* gene. Section of multiple alignment of the *Sult3a1* gene retrieved from the output result of ancestral sequences reconstruction step. From left to right, there is the species' name abbreviation, the chromosome's number, the strand in parenthesis, the position and the DNA sequence. The three dots represent parts of sequence not shown here. In bold and blue, are the exons described in *Caf* gene, with their number written above. The fragments of sequences that will be scanned are defined from the exon inference of the *Canis* gene, and are highlighted by a frame. The mis-position of nucleotides is highlighted in red. As consequence there is a frame shift which will not be detected, during the sequence scan.

In 2 cases our results are in line with published results, although GLADX provides less accurate conclusion. First, take the example of the *Sult3a1* gene described as a pseudogene in *Homo* [42]. Results given by GLADX show a pseudogenization that began in the LCA of *Catarrhini*. However, the mutations found in *Homo* are different to those described in the literature study. Exploiting manual expertise, we found that in contrast to other species, primates have orthologous to *Sult3a1 Mus* sequences without introns. We can deduce that the LCA of *Eutheria* should, in the most likely scenario, display the gene with introns. During the reconstruction step, only *Canis* gene was kept as primates out-group. Sequences without introns came out over-represented, and the reconstructed ancestral sequence of the LCA of *Eutheria* did not have introns. Despite this prediction hiccup, no error was introduced into the reconstructed exon sequences. As the gene was unknown in primates, to perform the scan at nucleotide level, the exons that were used in primates were modeled by the exon structure of the *Canis* gene. However, studied sequences are not perfectly aligned with the *Canis* gene. Indeed, one or two bases of studied exons that should be positioned in front of the end of *Canis* exons 2, 3 and 4, are positioned in front of introns (Figure 7). These misplaced bases cannot be observed by GLADX, which studies the sequences only opposite to known positions of reference exons. Consequently these missing bases cause a reading frameshift, and the mutations found by GLADX does not reflect reality of events occurring at nucleotide level during evolution of the gene. With a manual verification, we observe clearly that primates sequences contain numerous harmful mutations. Primates have pseudogenes, and pseudogenization seems to have begun at least in the LCA of *Catarrhini*. The pseudogenes in *Gorilla*, *Pongo*, *Pan* and *Homo* do not have introns and surely represent retroposon fixed at least since the LCA of *Catarrhini*. It is interesting to note that the original orthologs to *Mus Sult3a1* gene (ortholog from position) have been lost in these species, keeping only the retroposon which has become the unitary pseudogene in primates.

Another case in which GLADX results are less precise is the one of *Gulo* gene. It is an undergoing pseudogenization since at least the LCA of *Catarrhini*, and the bit of gene that remains in *Homo* contains a high number of mutations [5], [26], [27]. GLADX, via TBLASTN, detects the pseudogenized sequences in the *Catarrhini* species used, but their signals are too low to build gene phylogenies, and without phylogenetic confirmation of the orthology, GLADX concluded on a loss of *Gulo* in *Catarrhini*. The loss of *Gulo* function detected by GLADX is consistent with the high pseudogenization of the *Gulo* gene reported in other studies.

In contrast, the only result that was discordant with previously published information concerns the *2310042E22Rik Mus* gene. It has been described as a pseudogene in *Homo* [26], [27], but GLADX detected it as intact without harmful mutation. This gene is also saved in *Macaca,* is a pseudogene in *Pan,* and is lost in *Sus*. It appeared by duplication in the LCA of *Eutheria,* and a gene phylogeny of *Euteleostomi* phylum shows that the gene family exists at least since the LCA of *Tetrapoda*.

We then ran a second series of studies as a negative control testing a set of random genes, which are known to have an ortholog present in the human genome. In a majority of cases, the results agree with Ensembl annotation. When a gene is noted as present or pseudogene, the gene is effectively found present or pseudogene. In rare cases, GLADX results disagree with Ensembl as it found a potential functional gene that Ensembl has noted as pseudogene (as was the case for ortholog to *2310042E22Rik Mus* gene in *Homo*).

Numerous missing annotations and annotation errors are present in databases. The missing annotations are not a problem with GLADX because of the systematic annotation step which enables the finding of novel genes. During our analyses, it annotated several putative novel genes (*2310042E22Rik, Gpr33, Ctf2, Slc7a15, T2R2*) in different species (*Homo, Gorilla, Macaca, Mus, Bos, Equus, Ornithorhynchus, Oryzias*), which further demonstrates the tool’s ability to re-annotate sequences. Currently, the annotation errors as over-predictions are not detected automatically by GLADX. A review of the results is necessary to find suspicious cases. In our analyses, some results do not fit with already published results and/or give non-parsimonious results due to the presence of a gene in an unexpected species. After manual expertise, these outcomes seem be the result of over-predicted genes in the database. These suspicious annotations have been found for *Gorilla* in predictions of *Nradd, Nepn, Mup4, 1110012D08Rik and Slc7a15* genes, as well as for *Macaca* in predictions of *Slc7a15* gene (Text S2). They have been re-annotated by relaunching GLADX omitting their presence in database (Text S1, B). The relaunched studies seem to give better results that are more parsimonious, and these are the results being analyzed in this paper. The last kind of artifacts that can be encountered using GLADX, are those that occurred due to limits of the GLADX-integrated tools. As we found this type of problem for alignment in the case of *Sult3a1,* we also found a problem on the ancestral sequence reconstruction step in the case of *Slc7a15* (Text S2). Artifacts do not necessarily have a dramatic impact on the results; nevertheless unusual results must be interpreted cautiously.

## Discussion

We created the GLADX module implemented in the DAGOBAH framework as an attempt to totally automate the analysis of lineage-specific gene losses. The performed benchmark demonstrated the efficiency and power of GLADX to answer a majority of cases with details on gene losses or pseudogenization events. Its use has underlined the importance of the quality of genomic data and annotations available in databases. We have already seen that missing annotations are not a problem for GLADX which is able to annotate novel genes. As for mis-predicted and over-predicted genes in databases, they can be a real problem for analyses, as they not only give a false view of gene presence in the species concerned but also engender a mis-reconstruction of states of presence and absence in ancestors. The suspicious predictions can easily be detected upstream of the study by testing the intron size or the presence of initiator codon; or downstream by detection of particular and unusual patterns in the results of the phylogenetic tree produced as an output of the GLADX study. We have also seen that GLADX offers the possibility to easily and accurately re-annotate these selected suspicious annotations. GLADX represents an essential tool for analyzing the evolutionary history of orthologous genes groups, more specifically the gene family's retention in lineages. As GLADX is completely automated, it can be used at high-throughput to analyze a wide-range of gene datasets, with the additional strength that it can also be used on any *Metazoan* species dataset. As the number of complete genomes increases, the quality of analyses performed with GLADX will increasingly improve.

The fact that GLADX was developed in the DAGOBAH framework eases adding of new functionalities, and several new sources of data can be used. Moreover, it is possible to implement additional manual expertise. These two features can improve the quality of the results and their interpretation. For example, use of EST or mRNA databases can confirm the transcriptional activity of a pseudogene or saved gene. In the case of a pseudogene, the impact of any mutation detected by GLADX, on the transcript formed can be demonstrated. These databases can also be helpful to confirm any mutations detected, to analyze polymorphism and found potential mistakes, on the genomic sequences used. Furthermore, other databases may contain useful information such as those specialized in the sequence polymorphism [43], although outside of the human genome, which has been extensively researched, there is currently still insufficient data. Integration in GLADX of tools such as PAML [44] can highlight the kinds of selective pressures that sequences are subjected to. A pseudogene will be confirmed by neutral evolution, whereas a saved gene may be confirmed, and its behavior better understood, by positive or purifying selection. It is also possible to slightly modify GLADX to answer other questions or provide a different field view. In the near future, by integrating concepts linked to lateral gene transfers, it should be possible to create a specific version dedicated to studying bacterial genomes.

To conclude, in addition to GLADX being dedicated specifically to studying gene loss and pseudogenizations that are lineage-specific, other DAGOBAH agents are specialized in identifying through phylogenetic analyses, other event types, such as new protein architectures, duplications, and more. All these events are saved in an ontological database allowing to cross-check the evidences and deduce events of higher-level. Analyses based on evolutionary biology approaches allow to detect if several events occur at the same time, and precisely to show convergence and co-convergence. This brings to recognize links between environmental shifts and genetic and functional shifts, to better understand the evolutionary processes.

## Supporting Information

Figure S1 **Class Diagram of GLADX ontology.**
(TIF)

Table S1 **Summary of benchmarking results.**
(RTF)

Text S1 **Description of GLADX parameters.**
(RTF)

Text S2 **Analyses of artifacts.**
(RTF)

Text S3 **GLADX user’s manual.**
(PDF)
