# Supplementary figures and images for: GLADX: An Automated Approach to Analyze the Lineage-Specific Loss and Pseudogenization of Genes
Source: PLoS One. 2012 Jun 18;7(6):e38792. doi: 10.1371/journal.pone.0038792 (PMC3377690; doi:10.1371/journal.pone.0038792)

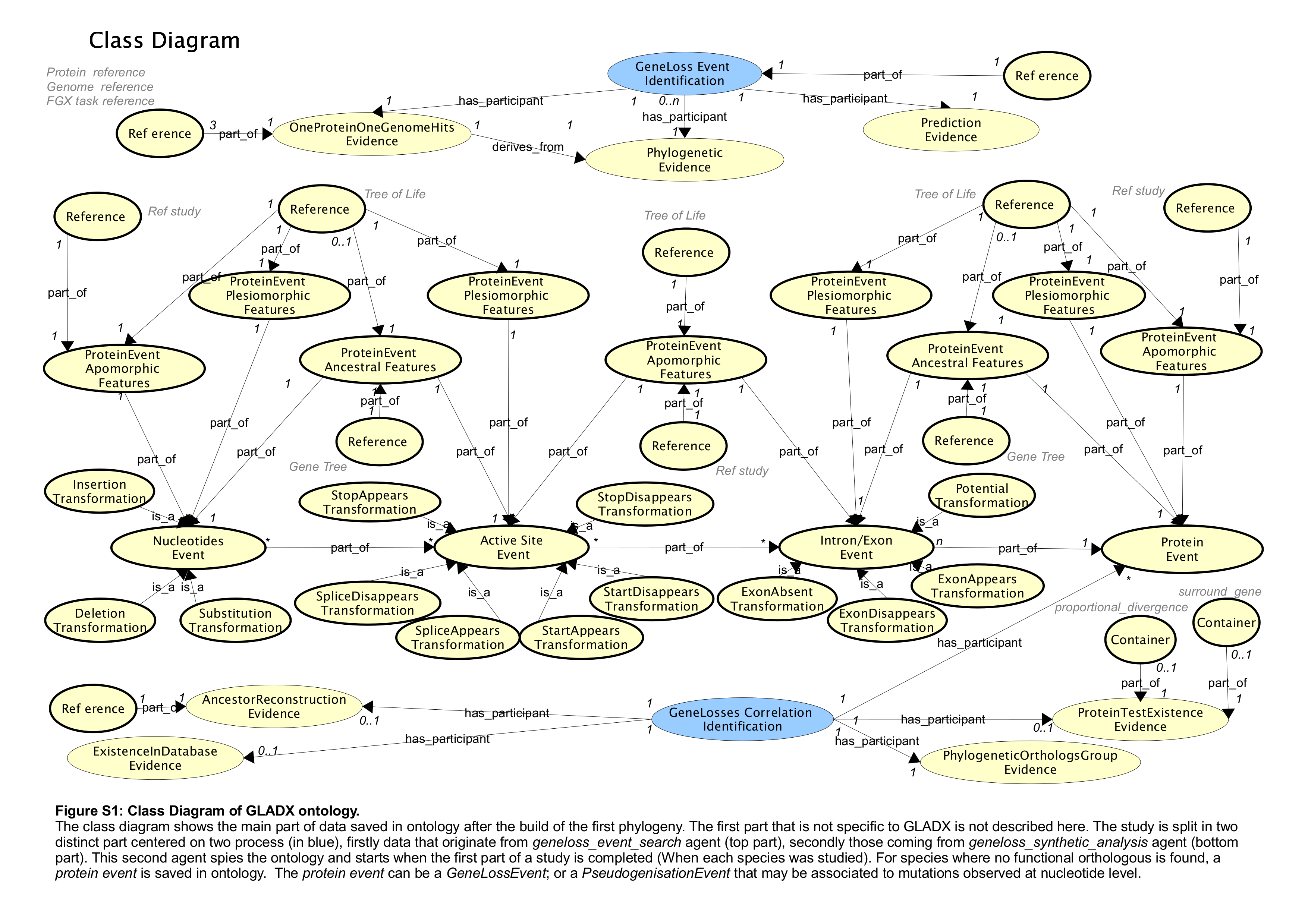

Supplement: Figure S1 — Class Diagram of GLADX ontology. (TIF) [file pone.0038792.s001.tif]
