# Supplementary material for: GLADX: An Automated Approach to Analyze the Lineage-Specific Loss and Pseudogenization of Genes
Source: PLoS One. 2012 Jun 18;7(6):e38792. doi: 10.1371/journal.pone.0038792 (PMC3377690; doi:10.1371/journal.pone.0038792)
Supplement: Table S1 — Summary of benchmarking results. (RTF) [file pone.0038792.s002.rtf]

Table S1: Summary of benchmarking results

Symbol	Gene name	Publication results	Species used for GLADX Study	Lineage studied	appea-rance	GLADX results	Artifacts	
2310042E22Rik	RIKEN cDNA 2310042E22 gene	(Zhu et al. 2007)(Zhang et al. 2010) Detected as pseudogene in Homo.	ENSMUSP00000100495	9347	9347 (D)	The gene is saved in Homo and Macaca, and lost in Sus. This is a pseudogene in Pan, where we observe a start codon mutation (ATG to CCG), an acceptor splice site mutation of exon 2 (TA to CA), a 5-bp insertion bringing a nonsense codon by frameshift and directly after, a 5-bp deletion restoring the initial reading frame. 		
Gulo	gulonolactone(-L) oxidase	(Nishikimi et al. 1994) A short piece of sequence was retrieved in Homo, within indels and nonsense codons.

(Zhu et al. 2007) Pseudogenization began before the Callithrix jacchus diverged from the human lineage by indels and nonsense codons.

(Zhang et al. 2010) Pseudogenization began in the LCA of Catarrhini.	ENSMUSP00000060912	117571	32523	A gene loss is observed in the LCA of Catarrhini. The gene is present at least since the LCA of Tetrapoda and seems to be a pseudogene in Xenopus. The gene is lost in Taeniopygia. Lot of mutation observed at genome level in Monodelphis and Ornithorhynchus allow concluding on a pseudogenization process in these species.		
Acyl3	Acyltransferase 3	(Zhu et al. 2007) Analysis at genome level shows a nonsense mutation (TGG to TGA) that inactivated Acyl3 after the divergence of Gorilla from the Homo lineage and before the Homo-Pan split.

(Zhang et al. 2010) Pseudogenization occurs after the divergence of Gorilla from the Homo lineage and before the Homo-Pan split.	ENSMUSP00000110749	7711***	7711	The gene is lost in Danio, Branchiostoma and in Neognathae lineage after the split with the LCA of Amniota, and is a pseudogene in Xenopus. It is found as a pseudogene in Homo, Pan and Pongo and seems to be intact in Gorilla. A splice site acceptor mutation (CT to CC) observed in the LCA of Hominidae may be the first event leading to the pseudogenization in Homo, Pan and Pongo. The gene is pseudogenized in Pongo independently by 3 nonsense codons that appeared by substitution (CAG to TAG; TAT to TAA; CGA to TGA) and an insertion of 4-bp at the end of the sequence, leading to a nonsense codon TGA by frameshift. A nonsense codon occurred in the LCA of Homo-Pan by substitution (TGG to TGA).  This last mutation has already been described in the literature.		
Uox	Urate oxidase	(Wu et al. 1989) They reported that the function of the enzyme has been lost in Homo and certain other primates. They observed two nonsense codons found in Homo that occurred by substitution (CGA to TGA; AGA to TGA). Pseudogenization seems to appear in Hominoid phyla after the divergence from Old World monkeys, but it's not clear whether it occurred after a common event or due to independent events.

(Oda et al. 2002) They studying the coding region and described a nonsense mutation shared by Homo, Pan, Gorilla and Pongo in exon 2 codon 33 that can explain the start of the pseudogenization process in the Hominidae phylum. They found two mutations in the LCA of Homininae, such as the acceptor signal mutation in exon 3 and a nonsense mutation at codon 187. They found in Gorilla a mutation in the initiation codon and a nonsense mutation. The Pongo has two independent nonsense mutations and the Pan one. In total they found 6 nonsense codons in Hominidae phylum. An independent pseudogenization also occurred in the Gibbon lineage. Indeed, they found a nonsense mutation,a single-base deletion and a single-base insertion shared by Hylobates lar, Hylobates agilis, Hylobates muelleri, Hylobates concolor and Hylobates syndactylus.

(Zhu et al. 2007) They found a pseudogenization of the Uox gene by nonsense mutation dated to before the Homo-Pan split.

(Zhang et al. 2010) They found a pseudogenization of Uox gene since the LCA of Hominidae.	ENSMUSP00000029837	117571	117571	All 6 nonsense codons already described in the Hominidae phylum and the acceptor site mutation of exon 3 were found. We found also a donor splice site mutation of exon 4 and an acceptor splice site mutation in exon 6 in Pongo. We also found a 7-bp insertion in the LCA of Homininae and a deletion of this insertion in Pan. The explanation for this indel is probably tied to allele sorting. To conclude, we found a pseudogenization of the Uox gene in Hominidae phyla, in agreement with other publications.		
Ctf2	Cardiotrophin 2	(Derouet et al. 2004) They reported the gene as intact in Pan. Homo has an 8-bp deletion in the putative third exon leading to shift and loss of the reading frame.

(Zhu et al. 2007) They found an 8-bp deletion engendering a pseudogene in Homo.

(Zhang et al. 2010) They detected the pseudogenization that occurred only in Homo.	ENSPTRP00000054644	117571	32523	The 8-bp deletion is observed and two nonsense codons inducted by this deletion are detected. We retrieve the gene in Gorilla and Macaca. GLADX was also able to show a gene loss in the Neognathae lineage after the split with the LCA of Amniota.		
Nradd	neurotrophin receptor associated death domain	(IHGSC 2004) They reported the pseudogene in Homo and in Pan. The Homo pseudogene contains one disruption.

(Zhu et al. 2007) They observed a nonsense mutation and an indel that allows dating the start of pseudogenization to the LCA of Hominidae.

(Zhang et al. 2010) They dated the start of pseudogenization to the LCA of Hominidae.	ENSMUSP00000035069	7711***	117571	The gene is lost in Ornithorhynchus and Taeniopygia. The gene started the pseudogenization in the LCA of Hominidae by a previously-described nonsense codon. Afterwards, there are independent mutations in Pongo, Gorilla, and Pan. In Pongo we observe two substitutions leading to nonsense codons (CGA to TGA; CAA to TAA). In Gorilla, there is a 1-bp deletion leading to the appearance of four nonsense codons by frameshift. In addition, we found a fifth nonsense codon exists, that appeared by addition of the frameshift and a substitution (TGG to TGA). In Pan there is a donor splice site mutation (GT to GC) in exon 2. No other mutations were found in Homo other than that found in the LCA of Hominidae. 	* ENSGGOP00000028206 (intron length 4, 2, 12)	
Nepn	nephrocan	(IHGSC 2004) They reported the pseudogene in Homo and Pan. The Homo pseudogene contains four disruptions.

(Zhu et al. 2007) They found a nonsense mutation and an indel that allow dating the pseudogenization to the LCA of Catarrhini.

(Zhang et al. 2010) They dated the start of pseudogenization to the LCA of Catarrhini.	ENSMUSP00000070130	7711***	7735	The gene is lost in Clupeocephala lineage after the split with the LCA of Euteleostomi and is also lost in Bos. GLADX save the gene in Branchiostoma. In the human lineage, the pseudogenization process began at least to the LCA of Catarrhini. We cannot observe the pseudogenization in Macaca as the gene is too extensively pseudogenized and is considered lost by GLADX. The gene composed of three exons was intact in the LCA of Eutheria, and we can see mutations from the LCA of Hominidae. The mutations observed in the LCA of Hominidae against the LCA of Catarrhini are two deletions (5 bp and 2 bp) in exon 2, a 1-bp insertion at the end of the sequence, 30 nonsense codons due to frameshifts, six of which are also linked to a substitution event. The indels engender a new reading frame for all the descendant species. In Pongo we observe the loss of the first and last exon. In the remaining exon there is a 1-bp deletion, which adds to the other frameshifts appearing in the LCA of Hominidae. There are six nonsense codons present in Pongo, all linked to the frameshifts, and among them two are also linked to substitution events (TAT to TAA; CGA to TGA). In the LCA of Homininae there are six nonsense mutations due to substitution events (TTA to TAA; TGG to TAG (*2) ; GAA to TAA; CAG to TAG; GGA to TGA). In Gorilla there are several indels in exon 2: two deletions (9 bp; 3 bp) and a 9-bp insertion that engender no frameshifts, and a 7-bp insertion that adds to the other frameshifts appearing in the LCA of Hominidae. The frameshifts and insertions give birth to two nonsense codons (--- to TAA (*2)). In Pan we found one nonsense codon by substitution (CGA to TGA).	* ENSGGOP00000023229 (intron length 1)	
Mup4	Major unitary protein 4	(Chamero et al. 2007) They reported that this is a unitary pseudogene only in Homo.

(Zhang et al. 2010) They found a mutation at the splicing donor (GT to AT) of exon2 of the Homo pseudogene. It is a pseudogene in Homo, and they observed an acceleration of the non-synonymous substitution rate in primates.	ENSMUSP00000095648	7711***	9347	The pseudogenization process began at least to the LCA of Catarrhini, by appearance of a nonsense codon (TAG) in the reading frame. No other mutations appear in Macaca. No harmful mutation appears in the LCA of Hominidae. In Pongo, a nonsense codon appears by substitution (CGA to TGA) in the penultimate exon. No harmful mutation appears in the LCA of Homininae. In Gorilla, there is a 1-bp insertion that induces seven nonsense codons. No new mutations appear in the LCA of Homo-Pan, or in Pan. On the other hand, a 41-bp insertion appears in Homo. The long insertion brings 3 new nonsense codons. We also found the previously-described donor splice site mutation of exon 2.	* ENSGGOP00000020262 (intron length 1)	
T2r2	Bitter taste receptor T2R2	(Go et al. 2005) They claimed the gene is polymorphic in terms of two-base deletion at codon position 160 in the Homo population.	ENSPTRP00000054801	117571	32523	The gene exists at least since the LCA of Tetrapoda, and was pseudogenized in the Phasianidae lineage after the split with the LCA of Neognathae, in Sus and Homo and was lost in Gallus and Macaca. Automated analysis at Homo genome level shows a pseudogenization due to a 2-bp deletion already described in other publications. This deletion brings six nonsense codons. GLADX also retrieved the gene in Mus, Bos and Equus, which have no harmful mutations.		
Tas2R134	taste receptor, type 2, member 134	(Go et al. 2005) They reported it as a pseudogene in Homo due to two nonsense mutations (CAG to TAG and GAA to TAA).

(Zhang et al. 2010) They found it pseudogenized in Homo only.	ENSPTRP00000054241	9347	9347 (D)	The gene appeared in the LCA of Eutheria and only became a pseudogene in Homo due to the two nonsense mutations already described in other publications.		
1110012D08Rik	RIKEN cDNA 1110012D08 gene	(Zhang et al. 2010) They detected the pseudogene in Homo.	ENSMUSP00000050451	9254	32524 (D)	Pseudogenization began in the LCA of Catarrhini by a 1-bp insertion event that induced 8 nonsense codons, one of which is also linked to a substitution event (CAC to TAA). In Macaca there is one mutation in an initiation codon (ATG to CTG) and a 10-bp deletion engendering seven nonsense codons, 4 of which are also linked to substitution events (TTA to TGA; CAG to TAG (*2); TAC to TAA). In the LCA of Hominidae there is no mutation. In Pongo there is a donor splice site mutation (GT to AT) and an initiation codon mutation (ATG to GTG). One nonsense codon appeared by substitution in the LCA of Homininae (CAA to TAA). In Gorilla there is a 1-bp deletion that induces one nonsense codon. In the LCA of Homo-Pan and in Homo there is no new harmful mutation. In Pan there is a 1-bp deletion that induces one nonsense codon.	* ENSGGOP00000021588 (introns length 4 and 11)	
Gpr33	G protein-coupled receptor 33	(IHGSC 2004) They reported a pseudogene in Homo by a disruptive element, and also a pseudogene in Pan.

(Zhu et al. 2007) They reported a pseudogene due to a nonsense codon that occurred only in Homo.

(Zhang et al. 2010) They reported a duplicated pseudogene in Homo.	ENSPTRP00000054852	32523	32523 (D)	The gene is pseudogenized in Homo by a nonsense codon due to a substitution (CGA to TGA). The gene is also pseudogenized in Pongo by a nonsense codon that appeared by substitution (CAT to TAA). The gene seems to be lost in Taeniopygia, Gallus, Monoddelphis and Equus. There is also a pseudogenization in Sus. Finally, the gene was saved in Ornithorhynchus.		
Slc7a15	solute carrier family 7 (cationic amino acid transporter, y+ system), member 15	(IHGSC 2004) They reported pseudogene in Homo and in Pan. The Homo pseudogene contains two undescribed disruptions.

(Zhu et al. 2007) They observed that the pseudogenization began before the Callithrix jacchus and Catarrhini lineage split by a nonsense codon.

(Zhang et al. 2010) They found disruptive mutations that gave rise to Homo unitary pseudogenes dated to before the Callithrix jacchus and Catarrhini lineage split.	ENSMUSP00000093548	7735***	7735	The gene is saved in Oryzias, pseudogenized in Branchiostoma and Catarrhini phylum, and lost in Xenopus, Neognathae phylum, Ornithorhynchus, Equus, Gasterosteus and Danio. Pseudogenization of the gene began in the LCA of Catarrhini by two deletions (1 bp * 2) and two insertions (8 bp; 319 bp). There is also an acceptor splice site mutation in the third exon (AG to CG) and nine nonsense codons. Among them, one is present in the inserted sequence, and two are linked to substitution events (CAG to TAG; CGA to TGA). In Macaca, the first exon disappeared; we also found one acceptor splice site mutation (CG to CA) in exon three, and no indels that induce nonsense codons. The LCA of Hominidae cannot be studied due to the reconstruction profile built by the “Ortheus” tool. In Pongo there is one exon disappearance, two deletions (1 bp; 4 bp), and 21 nonsense codons, 5 of which are linked to a substitution events (CGA to TGA; TGG to TGA; TGC to TGA; TGG to TAG; TAC to TAG). In the LCA of Homininae there is one donor splice site mutation in the first exon (GT to AT), a 5-bp deletion also in the first exon, and 14 nonsense codons. In Gorilla there is a 22-bp insertion that induces seven nonsense codons and one acceptor splice site mutation in the third exon (CG to TG). The LCA of Homo-Pan contains no mutation. In Pan there is a 1-bp deletion. In Homo there is a 1-bp insertion that induces three nonsense codons and one acceptor splice site mutation (CG to TG), as described in Gorilla. These common splice mutations between Homo and Gorilla may stem from allele sorting.	* ENSGGOP00000022534 (intons length: 1, 1, 14, 2)
ENSMMUP00000029429
(exon length: 5)
The study was relaunched with weak threshold allowing to analyzed Macaca at nucleotide level.
** Error in reconstruction that induces we cannot observe the first deletion.	
Sult3a1	sulfotransferase family 3A	(Freimuth et al. 2004) They observed four nonsense codons and a frameshift in the Homo pseudogene.	ENSMUSP00000090259	117571	117571	The pseudogenization process began at least in the LCA of Catarrhini. We cannot observe the pseudogenization in Macaca, since although the sequence is present in the genome, it is too pseudogenized to pass the identity threshold and thus be scanned at genome level. In the LCA of Hominidae, we found a 23-bp insertion and 10 nonsense codons, two of which are due to a substitution events (TTA to TGA; ATA to TGA). In Pongo we found two nonsense codons by substitution (TGG to TGA; AGA to TGA). There is no mutation in the LCA of Homininae. Gorilla has one nonsense codon that appeared by substitution (TGG to TGA) and is similar to that observed in Pongo but which seems to have appeared independently. There are no new mutations in the LCA of Homo-Pan or in Homo. In Pan we found a nonsense codon that appeared by substitution (CCA to TAG). 	** Error in alignment at the end of exons                     : Exon 2 it miss one base, at exon 3 and 4 it miss 2 bases.

Frame scanned not always well. It engenders observation of some mutation not implicated in pseudogenization processes.	

(D) = This symbol in “appearance” columns means that the orthologous group studied is occurred by a duplication which has been observed in phylogeny.
* = Some protein coding genes are described as intact in the Ensembl database but after manual inspection we observed that they seems to be over predictions. In these cases, the protein causing the problem was cited, and the study was relaunched with a parameter-set making it possible to avoid considering the protein as present. That allows re-annotating the gene by GLADX.
** = indicates tool-induced artifacts. An explanation is given for the kinds of problem engendered.
*** = In these cases we have implemented specifically in GLADX the capacity to use the Branchiostoma floriade species; this engenders the presence of a ancestral node with the taxid 7735.

Note: Some indels are not described here as they are multiples of three and do not seem to be essential to the pseudogenization analysis.
