# Supplementary material for: GLADX: An Automated Approach to Analyze the Lineage-Specific Loss and Pseudogenization of Genes
Source: PLoS One. 2012 Jun 18;7(6):e38792. doi: 10.1371/journal.pone.0038792 (PMC3377690; doi:10.1371/journal.pone.0038792)
Supplement: Text S1 — Description of GLADX parameters. (RTF) [file pone.0038792.s003.rtf]

Text S1: GLADX parameters

Numerous parameters are available to adjust the behavior of GLADX. Some are essential, such as species and used database, and mode of study (verification of putative lost genes or not). These parameters must be defined before analysis is launched. They are contained in an XML file accessible at:
/home/tower/TOWER_1.03/prod/DGH_2/dagobah.xml
The parameters of agents are defined between the following markups:
<engine-def>
<type>Agent_Name</type>
...
</engine-def>

A) Parameters defined in the fasta_protein_phylo agent:
“species_scope_for_phylogeny_study('9598,9606,9544,10116,10090,9601,9615,8090,9031,13616,7719,8364,99883,9593,9103,9913,9796,9823,9258,59729,69293,7955')” and
“species_scope_list_for_phylogeny_study(['9598','9606','9544','10116','10090','9601','9615','8090','9031','13616','7719','8364','99883','9593','9103','9913','9796','9823','9258','59729','69293','7955'])” are two identical species scopes (identified by taxid) with different formats employed to choose species used during the study. Phylogenies will be built with these species. The default value is that described above (22 species). 
“database('Path_database_used')” defines the path of the protein database used. The default path is '../AlgoTools/Blast/db/ensembl'.

B) Parameters defined in the geneloss_event_search agent:
“nucleotide_in_more_by_side(10000)” is the number of nucleotides taken on each side of a TBLASTN hit, to output a prediction (value must be identical to the genelosses_synthetic_analysis value). The default value is 10000.
“orthologs_group_mode(mode('TaxidAncestor'))” is the ortholog sequence analysis mode launched. There are two mode options: lineage or species. In the publication we speak only about the lineage mode that is appropriate to focus on the lineage-specific losses.
In lineage mode, GLADX searches the sub-tree having the TaxidAncestor ancestor as root and containing the reference given as input. All the sequences present in this subtree form an orthologous group. From this orthologous group it deduces the lineage-specific losses comparing the species present in the group to the species-set selected for the study. Note: An agent allowing analyzing systematically all nodes of the lineage leading to the input reference from the selected ancestor can be activated. => See G) section
In species mode, it searches in the phylogeny the species that have orthologs to the reference protein given as input until the TaxidAncestor ancestor and deduces losses comparing species that have an ortholog to the species-set selected for the study. This mode is less appropriate to analyze lineage-specific losses.
The default value is lineage('117571') that corresponds to a search of species that have no representative of a gene established at least since the last common ancestor of Euteleostomi.
“do_not_study_when_species_exist(['9606','9544'])” defines species that will stop the study if an ortholog exists in the first phylogeny. Should be empty if you want to analyse all the species where the gene is missing. If you need to concentrate on losses in a specific species, note its taxid here. If a database-described ortholog already exists for your species in the first phylogeny, there is no need to continue the study (to save your time). By default the value is empty.
“minimum_size_of_orthologs_group_for_begin_the_study(3)” is the minimum size of an ortholog group required in the first phylogeny to continue the study. The default value is 3.
“search_missing_cause_in_genome(choice)” defined if you want to use GLADX in complete mode to search for the genome of a species where orthologs are missing in the first phylogeny. Choice can be yes or no. If no is chosen, no verification of loss is made, and the results output come exclusively from analyses of the first phylogeny built from the chosen database (making the process much faster). The default value is yes.
“translate_in_gene_to_detect_ortholog_if_necessary(choice)” is defined when you have a tree of proteins that you want to translate into genes. This parameter allows comparing two ortholog protein groups using their respective genes. Choice can be yes or no. No is faster but a little less accurate. Indeed, different proteins of a same gene can be present in two trees. If no translation in gene is performed, the both protein will be not found as similar.
“force_to_analyse_this_species(['9593','9606'])” This parameter allows to annotate the list of selected species, even if an ortholog is found by phylogeny in the first step. By default the value is empty.

C) Parameters defined in the best_hit_fgx agent:
“max_nb_managed_hits('5')” is the number of hits retained from TBLASTN to continue the analysis. The higher this number is, more the GLADX analysis can be long. Indeed, GLADX tests one by one the orthology of hits. As long as no hit is found orthologous, GLADX continue to test the following hit. If there is no ortholog this step is only limited by the number of hit to be tested. Naturally it is possible that the number of hit found by BLAST can be inferior to the fixed value. The default value is 5.

D) Parameters defined in the genelosses_checkpoint_all_events_by_study agent:
“length_threshold(50)” is the minimum overlapping threshold between an orthologous sequence retrieved by GLADX and a known protein in order to continue the study at nucleotide level. The default value is 50.
“identity_threshold(50)” is the minimum identity threshold needed between an orthologous sequence retrieved by GLADX and a known protein to continue the study at nucleotide level. The default value is 50.
“identity_threshold_for_real_gene(70)” is the minimum identity threshold needed between known protein and used reference protein to be used in study at nucleotide level. The default value is 70.

E) Parameters defined in the genelosses_synthetic_analysis agent:
“nucleotide_in_more_by_side(10000)” is the number of nucleotides taken on each side of an orthologous gene to build an alignment with orthologs retrieved during the study. It is the step just before the reconstruction (The value must be identical to the geneloss_event_search value). The default value is 10000.

F) Parameters defined in the verify_prediction_existence agent:
When GLADX retrieves an ortholog, it systematically checks the database used to see whether there is an annotation on its position. Sometimes previously-described genes are present on the same area.
“overlap_threshold(50)” is the minimum overlap threshold in percentage for a previously-described gene in the database to consider that they are on the same position. The default value is 50.
“identity_threshold_to_conclude_gene_already_exist(70)” is the minimum identity threshold in percentage for a previously-described gene in the database overlapping the GLADX-retrieved ortholog sequence to be considered as the same prediction. The default value is 70.

G) Activation of the gladx_driver agent to automate the search of lineage-specific losses on all nodes:
Activation of this agent allows analyzing systematically the lineage-specific losses from all nodes available along the lineage leading to the input reference from the selected ancestor. 
“Targets(['9606'])” is a parameter defining the species concerned by lineage-specific loss, searched by GLADX. It allows focusing the search on the interest species. When no species are specified, GLADX searches all lineage-specific losses along the studied lineage. By default the value is empty.
The activation of agents is defined with these following markups:
<master>
<type>Agent_Name</type>
…
</master>
By default gladx_driver agent is deactivated by comment markups. To activate it, the comment markups of the gladx_driver agent must be removing, and the line of the orthologs_group_mode parameter of the geneloss_event_search agent must be commented.
Note: When new studies are performed with the gladx_driver agent, its orthologs_group_mode(lineage('TaxidAncestor')) parameter is used to define from which ancestor the study begin. While if the gladx_driver agent is launched after a first round of analysis with default mode, its orthologs_group_mode(lineage('TaxidAncestor')) parameter does not used. In this case, all the nodes of the lineage are analyzed from the ancestor that was defined at first round in the orthologs_group_mode(lineage('TaxidAncestor')) of geneloss_event_search agent.
