# Supplementary material for: GLADX: An Automated Approach to Analyze the Lineage-Specific Loss and Pseudogenization of Genes
Source: PLoS One. 2012 Jun 18;7(6):e38792. doi: 10.1371/journal.pone.0038792 (PMC3377690; doi:10.1371/journal.pone.0038792)
Supplement: Text S2 — Analyses of artifacts. (RTF) [file pone.0038792.s004.rtf]

Text S2: Analyses of artifacts:

Through our analyses using GLADX, we highlighted three types of artifacts: errors in genomic sequences, prediction errors, and errors due to the limits of the tools used.
Detection and analysis of mis-predictions and over-predictions present in the used database:
Here we will explain prediction errors, which appear to be the event the most widely encountered, and which we have not yet tackled. Among the 14 benchmarked cases of pseudogenes, the first round of GLADX analysis found 6 non-parsimonious cases in terms of number events, kinds of events, and location on the evolutionary timeline. In these cases, Homo, Pan, Pongo and sometimes Macaca had detected pseudogenes, but a gene was detected as present in Gorilla (Acyl3, Nradd, Nepn, Mup4, 1110012D08Rik, Slc7a15). Furthermore, 3 of these cases do not fit with published results, where Macaca gene (Slc7a15) and Gorilla genes (Nradd, Nepn, Slc7a15) should be pseudogene. After manual verification in the Ensembl database, we observed that small introns with less than 13 bases are present in 5 genes (Nradd, Nepn, Mup4, 1110012D08Rik, Slc7a15). Very few introns are known to be less than 20 bases in length. The smallest intron found in protist genes was 13-20 bases long [1]. The minimum CDS intron size observed in 2903 genes from 10 eukaryotes was 13 bases [2]. The minimum intron size in ESTs in a collection of fungi was 27 bases [3]. In the case of the Acyl3 case, there is just suspected short 8-base and 4-base-long exons in Gorilla. In the case of the Slc7a15 gene, there are suspected short exons in Macaca. There is some concern that very short exons might not be real exons. Short exons or introns may be the result of annotation correcting for apparent frameshifts. 
This evidence led us to suspect that these genes were over-predicted, with varying degrees of certainty according to clues. 
To verify these suspicions, we relaunched the analysis for each of the 6 cases, with a parameter that, depending on the case, allowed us to consider the Gorilla and/or Macaca gene as absent from the database. Using this parameter, found that a potential mutation may have occurred in these species, as well as in ancestors. The results obtained on the 5 genes with intron size problems show more parsimonious scenarios with a common mutation existing in ancestors. These results may point to the mis-prediction of introns in these Gorilla genes. In the case of Slc7a15 with suspect exon size in the Macaca gene, we again found a more parsimonious scenario, and confirmed the pseudogenization.
Based on these new results, we agree with the 3 cases of Gorilla and Macaca pseudogenization already described. Furthermore, in two cases (Mup4, 1110012D08Rik), the pseudogenization process was found to be older.
The analysis of the Acyl3 gene represents a particular case. Indeed, no pseudogenization has been reported in the Gorilla gene, and the gene described on Ensembl (ENSGGOP00000028123) showed no sequence problems except exons that were relatively short, at 8 bases and 5 bases long. Relaunching Acyl3 with Gorilla considered as absent from the database came up with the same result as in the previous study. Homo, Pan and Pongo are found as pseudogenes due to mutation. The nonsense codon that occurred by substitution was always present in the ancestor before the split between Homo and Pan and after Gorilla diverged from the human lineage. The differences in this relaunched study are that the LCAs of Hominidae and Homininae are scanned at genome level, as the functionality of the genes in these ancestors is considered as unknown. In the LCA of Hominidae, we observed an acceptor splice site mutation, but the gene is found as functional in Gorilla as there is no existing harmful mutation. No further mutation is shown in the gene of the LCA of Homininae. The Gorilla gene is consequently found as potentially functional (noted as saved by the tool) as there is no harmful mutation in the Gorilla sequence or its ancestral sequences (Hominidae and Homininae). The splice site mutation in the LCA of Hominidae seems to be the first event leading to pseudogenization. Our results are in agreement with Ensembl and with previously published results [4] about functionality of the Gorilla gene. This splice site mutation in the LCA of Hominidae may have triggered the pseudogenization process in Homo, Pan and Pongo, but Gorilla seems to have found a way to keep its gene intact. Our results show several missing exons in Gorilla, some of which seem to be in sequencing gaps. The results exposed need to be treated with caution, as there may be mutation in these missing exons. 

Problem encountered with reconstruction of ancestral sequences:

The GLADX analysis of Slc7a15 highlights some nonsense codons, with no substitution or frameshift events observed. We searched for an explanation by manually running an analysis of the ancestral sequence reconstruction product and its alignment. We found one deletion of 1 base at the start of the primates genes that was absent in genes of other species. The gene in the LCA of Eutheria should have the gene without this deletion, but there is an indel error in the reconstruction, and the deletion has been ascended in the LCA of Eutheria. Consequently, when we compared the gene of the LCA of Catarrhini against that of the LCA of Eutheria, the deletion event was not seen. GLADX cannot observe these indels but is able to observe the event engendered. We also observed that a substitution leading to the first nonsense codon (linked to the frameshift) also went undetected. Indeed, focusing on the indel, a nonsense codon normally only present in primates was ascended in the Eutheria ancestral sequence reconstructed. The codon concerned in Canis is the CAG in position 17. It becomes a TGA in primates. Slc7a15 is an example that underlines the limits of tools integrated in GLADX. Here it engenders no error, but some information is missed.

1.	Russell CB, Fraga D, Hinrichsen RD (1994). Extremely short 20–33 nucleotide introns are the standard length in Paramecium tetraurelia. Nucleic Acids Research, 22(7), 1221-1225.
2.	Deutsch M, Long M (1999). Intron-exon structures of eukaryotic model organisms. Nucleic Acids Research, 27(15), 3219-28. 
3.	Kupfer DM, Buchanan KL, Lai H, Zhu H, Dyer DW et al. (2004). Introns and splicing elements of five diverse fungi. Eukaryotic Cell, 3(5), 1088-1100. 
4.	Zhu J, Sanborn JZ, Diekhans M, Lowe CB, Pringle TH et al (2007). Comparative genomics search for losses of long-established genes on the human lineage. PLoS Computational Biology, 3(12), e247.
