# Supplementary material for: GLADX: An Automated Approach to Analyze the Lineage-Specific Loss and Pseudogenization of Genes
Source: PLoS One. 2012 Jun 18;7(6):e38792. doi: 10.1371/journal.pone.0038792 (PMC3377690; doi:10.1371/journal.pone.0038792)
Supplement: Text S3 — GLADX user’s manual. (PDF) [file pone.0038792.s005.pdf]

Gene Loss Analyzer DAGOBAH eXtension (GLADX) User's Manual

EBM (Evolutionary Biology and Modeling) Laboratory - UMR 7353

Aix-Marseille University - France

Download the (VirtualBox) GLADX image... (~17Go)

Link to benchmark analysis of 14 reported cases (Currently only available for identified users)

The current GLADX version enables using 22 species from Ensembl v57: Bos taurus, Canis familiaris, Ciona intestinalis;, Danio rerio, Equus caballus, Gasterosteus aculeatus, Gallus Gallus, Gorilla gorilla, Homo Sapien, Monodelphis Domestica, Meleagris Gallopavo, Macaca Mulatta, Mus Musculus, Ornithorhynchus anatinus, Oryzias latipes, Pan Troglodytes, Pongo Pygmaeus Abelii, Rattus norvegicus, Sus scrofa, Taeniopygia guttata, Tetraodon nigroviridis, Xenopus Tropicalis.

GLADX corresponds to a set of agents. The sources are freely available and could be retrieved in the */home/tower/TOWER_1.03/prod/DGH_2/src/* **directory.**

The distributed GLADX version could easily be modified (as described in the procedure below) in order to increase the number of studied species. Thus, GLADX can also function with any species present in Ensembl from the version 48 to 58 (included) giving the option to work with 51 different species.

Table of contents

- Introduction
  - Technical requirements
- GLADX launch
- Choice of phylum and species studied
- Produced data and results
- How to add new species retrieved from Ensembl ?
  - 1) Install proteome and/or genome
  - 2) Create the tree topology
    - 2.1) Database modifications
    - 2.2) Advise the length of branches
- Can I use an other kind of protein database ?
- GLADX parameters

Introduction

GLADX is a module included in a software application: DAGOBAH (Gouret et al., 2011). According to its name (Gene Loss Analyzer DAGOBAH eXtension), it is dedicated to gene losses and pseudogenizations automatic detection and analysis.

DAGOBAH relies on other relevant software tools (FIGENIX (Gouret et al., 2005), PhyloPattern (Gouret et al., 2009), IODA (In press, http://ioda.univ-provence.fr)).

All these components form the lab's bioinformatic software platform, called: T.O.W.E.R (Tools Operating With Evolutive Resources). GLADX work in the TOWER framework.

For us and for external users, TOWER is now very complex to install, because one has to deploy many software components, many bioinformatics binaries, many databases and many genomic data.

So we chose the virtualization strategy, that means the installation of all TOWER's components, on a virtual machine image. Several image instances can be started, as virtual computers, on computers which disposes of a virtualisation software like VirtualBox, VMWare, ... and on Clouds.

Technical requirements

We decided to build an Ubuntu 11.04, 64-bit image on VirtualBox 4.1.2 (Oracle TM). Therefore, this image will work efficiently on 64-bit architecture host computers.

To run one image of TOWER, we recommend using a four cores workstation, with 4Go of RAM (minimal configuration). Our image is configured, as a default, to run with eight cores and with 8 Go (current workstation producing our tests).

Please, note that hardware virtualization technology has to be activated on the host computers. (VT-X for Intel, AMD-V for AMD) in order to obtain most advantageous performances.

Warning: the hyperthreading technology with OpenMPI, that is a software layer used to exploit parallel computing with bioinformatics softwares like Tree-Puzzle, ClustalW, is not recommended because of reported bugs.

Note: the eight cores are not strictly required for the image, users could modify the scripts as below:

- in /home/tower/TOWER_1.03/prod/FGX_API/scripts/puzzle_cmd_perl, change “-np 8” by “-np X”, where X is the number of cores you want to use
- same procedure for: /home/tower/TOWER_1.03/prod/FGX_API/scripts/clustalw_cmd
- same procedure for: /home/tower/TOWER_1.03/prod/FGX_API/pipelines/Templates/__CassiopePhylo+M__, replace “-a 8” in block:

```
<nodeRef>blast</nodeRef>
<parameterAssignation>
<parameterName>options</parameterName>
<parameterValue>-a 8</parameterValue>
```

About the network configuration of the image, the NAT mode was set as a default. This mode doesn't allow 'ssh' access but it is very much faster than Bridge mode.

Images can be run with or without X Window GUI (quite slow in the emulation). In NAT mode, RDP clients can be used to access to a non graphical image. In Bridge mode, one can use ssh.

Important : the *tower* user has *t0wer* as password in ssh or graphical mode.

GLADX image is downloadable on the following link: GLADX image. First uncompress it, then add it to VirtualBox with the GUI or with "vboxmanage -registervm ...." command.

GLADX launch

GLADX is started on boot of the image (with VirtualBox). In order to start a gene study with GLADX, one just has to deposit one or several FASTA files (amino acid sequences) in the following directory: */home/tower/GLADX_DATA*

The FASTA files require to be named as follows: *EnsemblProteinSequenceName.Taxid.fasta*

This file must contain a sequence in FASTA format with an header in the following format:

>lcl|EnsemblProteinSequenceName|Taxid|Species~Name|OptionalyADescription

corresponding in this actual example:

>lcl|ENSP00000375415|9606|HOMO~SAPIENS|

A golden dataset of 14 FASTA files corresponding to the cases reported at http://ioda.univ-provence.fr/ is available in the directory /home/tower/Examples/Fastas.

Additional options:

Users can deactivate automatic start of GLADX on boot of the image by commenting with a '#' the line 'su tower -c /home/tower/TOWER_1.03/prod/DGH_2/start' of the file '/etc/rc.local'.

In this configuration you need to launch DAGOBAH using the command 'start' in a Terminal from the current directory */home/tower/TOWER_1.03/prod/DGH_2.*

To stop DAGOBAH you just need to press 'CTRL+C' or alternatively to kill the process.

Choice of phylum and species studied

The default parameters of GLADX allow to analyze lineage-specific gene losses in Euteleostomi (or from the closest ancestor in leaves direction whether the gene is appeared later) by studying the orthologous group containing the protein reference given as input. By default 22 chordates species are used with the topology described below:

Tree of life of 22 species implemented in GLADX

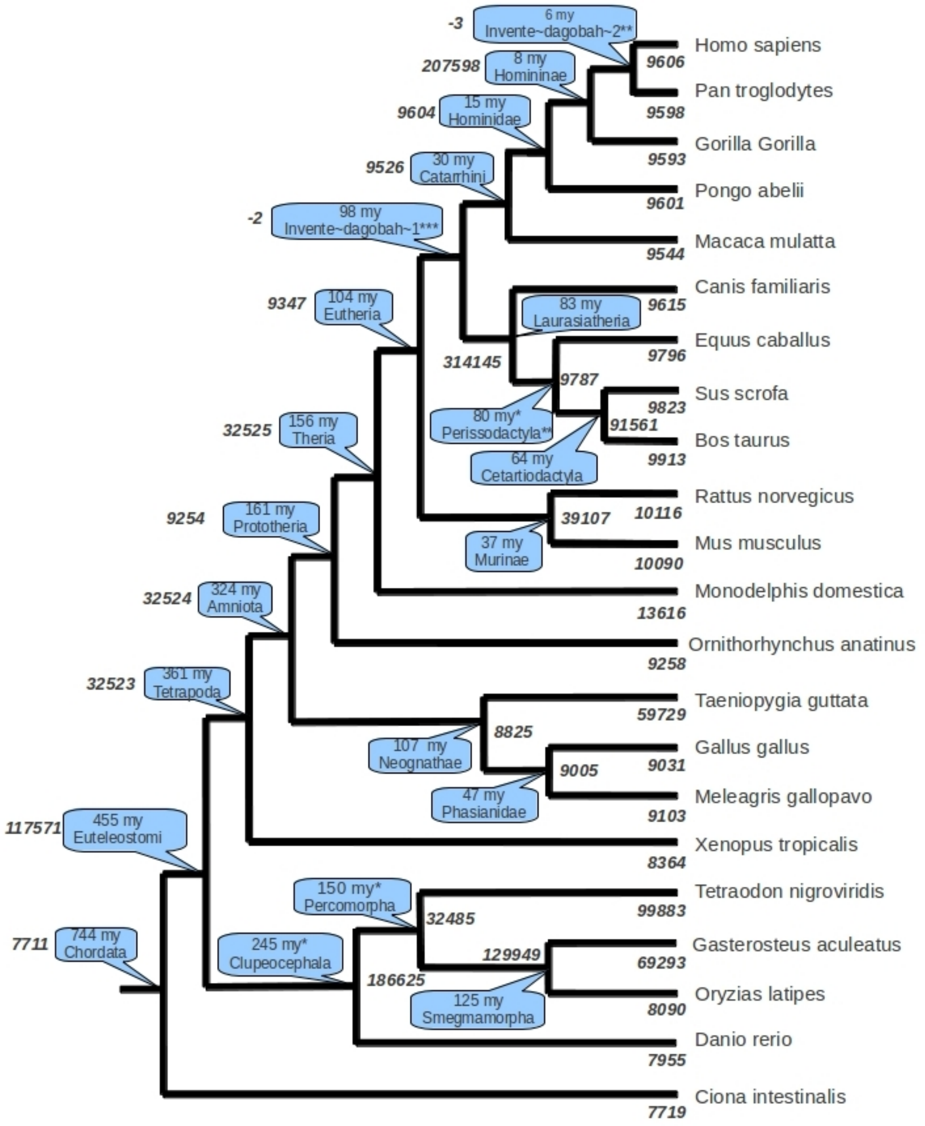

Date of speciations: They come from *TimeTree* (http://www.timetree.org/) and are displayed by million of years (my). When the date was not available it is indicates by "*" and we have chose a coherent date.

Species and ancestor names: The names are taken from *NCBI taxonomy*. When a common ancestor remain unclear and it exists a rake in NCBI, we have noted it by "**". In these case we have decided one topology and chose a name for the ancestors. When an ancestor name does not exist due to a topology incoherent between our choice and the NCBI topology, there is noted "***".

Taxid identifiers: For each leaves and ancestral nodes it exists an unique taxonomic identifier. These identifiers are numbers that are noted close to leaves or ancestral nodes. To follow our topology two ancestor names were invented and consequently no taxid exist, so we have tied to the ancestral species a negative unique identifier.

On these 22 species, by default 21 species of Euteleostomi are studied because the 'orthologs_group_mode' parameter defined in the */home/tower/TOWER_1.03/prod/DGH_2/dagobah.xml* file is parameterized to analyze losses

in Euteleostomi (taxid = 117571) in ***lineage*** mode. However, the analysis of largest phylum such as Chordates (including *Ciona*) is conceivable by using the taxid 7711. In the contrary, smallest phylum could be studied by using the taxid of any ancestor described in the figure 1. The number of species studied in a phylum may be modified by choosing among the 22 species those kept in the scope parameters (*species_scope_for_phylogeny_study* & *species_scope_list_for_phylogeny_study*).

Produced data and results

Results are automatically produced as .report files and databases contents. Report files can be easily read by our "user friendly" viewer FGXView (/home/TOWER/FGXView). The most important result is the final species tree of species-set in which all the results are pinpointed.

Databases contents are of two kinds:

- FIGENIX results produced on a SGBDR PostgreSQL in the database: *figenix_db*

- DAGOBAH results produced as an ontological database (see supplement 1), that relies also on a SGBDR PostgreSQL database: *dagobah_db*.

Note that these databases can be deployed on our IODA web site through collaborations.

1) Manipulate databases (*figenix_db* and *dagobah_db*) in SQL:

To manipulate these databases, please use the following commands in a Terminal:

- To backup the database in SQL format: pg_dump *DatabaseName* -f *SavingFileName*
- To delete a database: dropdb *DatabaseName*
- To create a database: createdb *DatabaseName*
- To install a new database: psql *DatabaseName* -f *DatabaseToInstall*

Note: *DatabaseToInstall* may be a database saved earlier or a clean figenix_db or dagobah_db database available in the directory: /home/tower/Examples/Databases

Warning: There is an incompatibility with the SGBDR PostgreSQL when the version >8.2 is used (we used 8.4). When a new database is created, before database installation you must be connected to the database (as postgres user, “sudo su postgres”, then “psql *DatabaseName*”) and past the text present in the */home/tower/jena_with_postgres_higher_than_8.2* file.

2) Manipulate ontological database (only *dagobah_db*) in OWL:

The ontological results can be exploited by *Protege* software and exported in “.OWL” files.

A script named *clear_with_file* is available at the directory */home/tower/TOWER_1.03/prod/DGH_2.*

It allows to delete a database and to install a new database from an .OWL file. In this case, you need to be in the *DGH_2* directory and use the command in a Terminal like this:

clear_with_file dagobah_model *CompletePathOfTheDatabaseName.owl*

Example to install a *dagobah_db* ontological database empty:

clear_with_file dagobah_model */home/tower/Examples/Databases/dagobah_db_empty.owl*

To backup the *dagobah_db* database in owl without use of *Protege* you need to be in the directory *DGH_2* and launch the following command:

owldump *NameOfBackup.owl*

How to add new species retrieved from Ensembl ?

The current GLADX version enables using 22 species, but more species can be used by some manipulation.

1) Install proteome and/or genome

- Genomes are required when you use GLADX in "complete mode" (parameter 'search_missing_cause_in_genome' in the *dagobah.xml* file). The genomes of species already present are in the directory */home/tower/TOWER_1.03/prod/FGX_API/GenomicDB/ensembl_dna/*. To add new species you need to add the formatted (command *formatdb* in Blast package) genome in this directory. You need also to add the path of the formatted file containing the DNA in the file */home/tower/TOWER_1.03/prod/DGH_2/src/project_specific.pl* like this: "species_dna_database('*Taxid*', '*PathOfTheSpeciesDNAFile*')."

- The proteomes of species already present are in the file */home/tower/TOWER_1.03/prod/FGX_API/GenomicDB/ensembl*. To add new species you need to add the proteome in this file and re-formatted it (command *formatdb* in Blast package).

Note: When you add a new proteomes or/and a new genomes, you need to format the FASTA headers as follows :

>lcl|ENSP00000375415 |9606|HOMO~SAPIENS|

corresponding to

>lcl|*SequenceName* |*Taxid*|*Species~Name*|*OptionalyADescription*

2) Modify the tree topology

The binary species tree defined in GLADX needs to contain the species chosen for analyses.

2.1) Database modifications

The tree topology of species is provided into FIGENIX database (called figenix_db) in the *dagobahtreeoflife* table. The topology is described branch by branch where each taxid is linked to its parent taxid and a description of its rank (*class* if it is an ancestral node, *species* if it is a leaf). An ancestral node must be linked to two taxid corresponding to their child nodes.

Warning: if you add new species that are outgroup of species already present: the farthest ancestor must always be linked to the ghost root taxid *1*.

2.2) Advise the length of branches

The length of branches of the species tree topology is defined in the file */home/tower/TOWER_1.03/prod/DGH_2/src/project_specific.pl* as follows:

“tof_branch_length_to_node(*'taxid','branch_length'*).”

You need to add all the new branch lengths.

Note: When you change the version of Ensembl data you must change the value of the 'maxEnsemblBuildNumber' parameter with the corresponding Ensembl version. This parameter is in the file */home/tower/TOWER_1.03/prod/DGH_2/ENSJHelper.properties*

Particular Case: If you add new species that are outgroup of chordate you need to change the taxid of the far ancestor of the new tree topology in the */home/tower/TOWER_1.03/prod/DGH_2/src/project_specific.pl* file replaced the taxid defined in “*dagobah_treeoflife_database_root*('7711').”

Can I use an other kind of protein database ?

Yes but only in “***simple*** mode”. To use the ***simple*** mode you need to modify the *dagobah.xml* file available at this path */home/tower/TOWER_1.03/prod/DGH_2/.*

1. <u>change the mode as described below:</u>

   search_missing_cause_in_genome('no')

2. <u>change the path to your new database</u>

   database('*Path_database_used*')

GLADX parameters

Numerous parameters are available to adjust the behaviour of GLADX. Some are essential, such as species and used database, ortholog detection mode (from the used reference sequence, or from its ortholog the most exterior depending to the selected phylum), and mode

of study (verification of putative lost genes or not). These parameters must be defined before analysis is launched. They are contained in an XML file accessible at:

/home/tower/TOWER_1.03/prod/DGH_2/dagobah.xml

The parameters of agents are defined between the following markups:

<engine-def>

<type>*Agent_Name</type>*

...

</engine-def>

A) Parameters defined in the fasta_protein_phylo agent:

"**species_scope_for_phylogeny_study(*'9598,9606,9544,10116,10090,9601,9615,8090,9031,13616,7719,8364,99883,9593,9103,9913,9796,9823,9258,59729,69293,7955'*)**" and

"**species_scope_list_for_phylogeny_study([*'9598','9606','9544','10116','10090','9601','9615','8090','9031','13616','7719','8364','99883','9593','9103','9913','9796','9823','9258','59729','69293','7955'*])**" are two identical species scopes (identified by taxid) with different formats employed to choose species used during the study. Phylogenies will be built with these species. The default value is that described above (22 species).

"**database('*Path_database_used*')**" defines the path of the protein database used. The default path is '../AlgoTools/Blast/db/ensembl'.

B) Parameters defined in the geneloss_event_search agent:

"**nucleotide_in_more_by_side(*10000*)**" is the number of nucleotides taken on each side of a TBLASTN hit, to output a prediction (value must be identical to the genelosses_synthetic_analysis value). The default value is 10000.

"**orthologs_group_mode(*mode*('*TaxidAncestor*'))**" is the ortholog sequence analysis mode launched. There are two ***mode*** options: ***lineage*** or ***species*** (cf. article).

In ***lineage*** mode, GLADX searches the sub-tree having the ***TaxidAncestor*** ancestor as root and containing the reference given as input. All the sequences present in this subtree form an orthologous group. From this orthologous group it deduces the lineage-specific losses comparing the species present in the group to the species-set selected for the study. **/!\ An agent allowing to analyze systematically all nodes of the lineage leading to the input reference from the selected ancestor can be activated. => see G) section**

In ***species*** mode, it searches in the phylogeny the species that have orthologs to the reference protein given as input until the TaxidAncestor ancestor and deduces losses comparing species that have an ortholog to the species-set selected for the study.

The default value is *lineage('117571')* that corresponds to a search of species that have no representative of a gene established at least since the last common ancestor of *Euteleostomi*.

"**do_not_study_when_species_exist([*'9606','9544'*])**" defines species that will stop the study if an ortholog exists in the first phylogeny. Should be empty if you want to analyse all the

species where the gene is missing. If you need to concentrate on losses in a specific species, note its taxid here. If a database-described ortholog already exists for your species in the first phylogeny, there is no need to continue the study (to save your time). By default the value is empty.

"**minimum_size_of_orthologs_group_for_begin_the_study(*3*)**" is the minimum size of an ortholog group required in the first phylogeny to continue the study. The default value is 3.

"**search_missing_cause_in_genome(*choice*)**" defined if you want to use GLADX in complete mode to search for the genome of a species where orthologs are missing in the first phylogeny. ***Choice*** can be **yes** or **no**. If **no** is chosen, no verification of loss is made, and the results output come exclusively from analyses of the first phylogeny built from the chosen database (making the process much faster). The default value is **yes**.

"**translate_in_gene_to_detect_ortholog_if_necessary(*choice*)**" is defined when you have a tree of proteins that you want to translate into genes. Allows comparing two ortholog groups of a gene or two ortholog groups of a protein. *Choice* can be **yes** or **no**. **No** is faster but a little less precise.

"**force_to_analyse_this_species([*'9593','9606'*])**" This parameter allow to annotate the list of selected species, even if an ortholog is found by phylogeny in the first step. By default the value is empty.

C) Parameters defined in the best_hit_fgx agent:

"**max_nb_managed_hits('*5*')**" is the number of hits retained from TBLASTN to continue the analysis. More this number is high, more the GLADX analysis can be long when putative tested homologous sequences are not orthologous. The default value is 5.

D) Parameters defined in the genelosses_checkpoint_all_events_by_study agent:

"**length_threshold(50)**" is the minimum overlapping threshold between an orthologous sequence retrieved by GLADX and a known protein in order to continue the study at nucleotide level. The default value is 50.

"**identity_threshold(50)**" is the minimum identity threshold needed between an orthologous sequence retrieved by GLADX and a known protein to continue the study at nucleotide level. The default value is 50.

"**identity_threshold_for_real_gene(70)**" is the minimum identity threshold needed between known protein and used reference protein to be used in study at nucleotide level. The default value is 70.

E) Parameters defined in the genelosses_synthetic_analysis agent:

"**nucleotide_in_more_by_side(*10000*)**" is the number of nucleotides taken on each side of an orthologous gene to build an alignment with orthologs retrieved during the study. It is the step just before the reconstruction (The value must be identical to the geneloss_event_search value). The default value is 10000.

F) Parameters defined in the verify_prediction_existence agent:

When GLADX retrieves an ortholog, it systematically checks the database used to see whether there is an annotation on its position. Sometimes previously-described genes are present on the same area.

"**overlap_threshold(*50*)**" is the minimum overlap threshold in percentage for a previously-described gene in the database to consider that they are on the same position. The default value is 50.

"**identity_threshold_to_conclude_gene_already_exist(*70*)**" is the minimum identity threshold in percentage for a previously-described gene in the database overlapping the GLADX-retrieved ortholog sequence to be considered as the same prediction. The default value is 70.

G) Activation of the *gladx_driver* agent to automate the search of lineage-specific losses on all nodes:

Activation of this agent allows to analyze systematically the lineage-specific losses from all nodes available along the lineage leading to the input reference from the selected ancestor.

"**Targets([*'9606'*])**" is a parameter defining the species concerned by lineage-specific loss, searched by GLADX. It allows to focus the search on the interest species. When no species are specified, GLADX searches all lineage-specific losses along the studied lineage. By default the value is empty.

The activation of agents are defined with these following markups:

<master>

<type>Agent_Name</type>

…

</master>

By default *gladx_driver* agent is deactivated by comment markups. To activate it, the comment markups of the *gladx_driver* agent must be removing, and the line of the **orthologs_group_mode** parameter of the *geneloss_event_search* agent must be commented.

Note: When new studies are performed with the *gladx_driver* agent, its **orthologs_group_mode(*lineage*('*TaxidAncestor*'))** parameter is used to define from which ancestor the study begin. While if the *gladx_driver* agent is launched after a first round of analysis with default mode, its **orthologs_group_mode(*lineage*('*TaxidAncestor*'))** parameter does not used. In this case, all the nodes of the lineage are analyzed from the ancestor that was defined at first round in the **orthologs_group_mode(*lineage*('*TaxidAncestor*'))** of geneloss_event_search agent.
